# Sex differences in Alzheimer’s disease CSF biomarkers and their association with Aβ pathology on PET in cognitively unimpaired individuals

**DOI:** 10.1186/s13195-025-01844-1

**Published:** 2025-10-30

**Authors:** Marta Milà-Alomà, Carol Van Hulle, Anna Brugulat-Serrat, Margot Casals Brodú, Armand González-Escalante, Gonzalo Sánchez-Benavides, Mahnaz Shekari, Laura Castro-Aldrete, Carolina Minguillón, Julie Novakova Martinkova, Maria Carmela Tartaglia, Clara Quijano-Rubio, Gwendlyn Kollmorgen, Annemarie Schumacher Dimech, Davide Cirillo, Frances-Catherine Quevenco, M. Florencia Iulita, Karine Fauria, Juan Domingo Gispert, Maria Teresa Ferretti, Antonella Santuccione Chadha, Sterling C. Johnson, Marc Suárez-Calvet

**Affiliations:** 1 Barcelonaβeta Brain Research Center, Pasqual Maragall Foundation, Barcelona, Spain; 2 Northern California Institute for Research and Education, San Francisco, CA USA; 3 Department of Radiology and Biomedical Imaging, University of California San Francisco, San Francisco, CA USA; 4 Wisconsin Alzheimer’s Disease Research Center, School of Medicine and Public Health, University of Wisconsin-Madison, Madison, WI USA; 5 Hospital del Mar Research Institute, Barcelona, Spain; 6 Centro de Investigación Biomédica en Red de Fragilidad y Envejecimiento Saludable (CIBERFES), Madrid, Spain; 7 Global Brain Health Institute, San Francisco, CA USA; 8 Universitat Pompeu Fabra, Barcelona, Spain; 9 Women’s Brain Foundation, Basel, Switzerland; 10 Memory Clinic, Department of Neurology, Second Faculty of Medicine, Charles University and Motol University Hospital, Prague, Czech Republic; 11 Tanz Centre for Research in Neurodegenerative Diseases, University of Toronto, Toronto, ON Canada; 12 Memory Clinic, Krembil Brain Institute, University Health Network, Toronto, ON Canada; 13 Roche Diagnostics International Ltd, Rotkreuz, Switzerland; 14 Roche Diagnostics GmbH, Penzberg, Germany; 15 Department of Health Sciences and Medicine, University of Lucerne, Lucerne, Switzerland; 16 Barcelona Supercomputing Center, Life Sciences Department & Bioinfo4Women, Barcelona, Spain; 17 Center for Alzheimer Research, Karolinska Institute, Solna, Sweden; 18 Neurology Department, Hospital del Mar, Barcelona, Spain

**Keywords:** Cerebrospinal fluid, Biomarkers, Preclinical, Alzheimer’s disease, Sex

## Abstract

**Background:**

Alzheimer’s disease (AD) exhibits sex differences in prevalence, symptoms and risk factors. Understanding the effect of sex in AD cerebrospinal fluid (CSF) biomarkers and their association with amyloid-beta (Aβ) pathology in preclinical stages have important implications for their use in prevention trials. The objective of this study was to examine sex differences in core AD CSF biomarkers used in early diagnosis and prevention trials, as well as in CSF biomarkers reflecting downstream pathophysiological mechanisms, and in their associations with Aβ pathology as measured by Positron Emission Tomography (PET).

**Methods:**

Cognitively Unimpaired (CU) participants from the ALFA + (N = 400) and the WRAP/WADRC (N = 548) cohorts were included in the study. CSF biomarkers for core AD pathology (Aβ42, Aβ42/40, p-tau181/Aβ42, p-tau181, p-tau217 and p-tau231), neurodegeneration (NfL, t-tau), synaptic dysfunction (neurogranin, GAP-43, SNAP25, synaptotagmin-1, α-synuclein), glial reactivity (GFAP, S100B, sTREM2, YKL-40), neuroinflammation (IL-6, MCP-1), and vascular dysregulation (sICAM-1, sVCAM-1) were measured. Participants underwent Aβ PET at baseline and follow-up visit. We used Analyses of Covariance (ANCOVA) to evaluate sex differences in CSF biomarker levels and performed sex-stratified Receiver-Operating Characteristic (ROC) analyses to test their performance to identify Aβ PET-positive individuals. Additionally, we run linear regression models to study the modifying effect of sex on the association of baseline CSF biomarkers with cross-sectional and longitudinal Aβ PET uptake.

**Results:**

Men had higher CSF NfL, glial reactivity and vascular dysregulation biomarkers (Cohen’s *d* ranging from -0.22 to -0.44, *P* < 0.05), and lower synaptic biomarkers (Cohen’s *d* ranging from 0.18 to 0.30, *P* < 0.05) compared to women at baseline**.** There were no sex differences in the core AD CSF biomarkers’ performance to identify Aβ PET-positive individuals (DeLong's test *P* values > 0.05), with CSF p-tau181/Aβ42 and p-tau217 showing the highest performance in both sexes (Areas Under the Curve (AUCs) ranging from 87.1 to 96.3). However, sex modified the associations of baseline CSF biomarkers with Aβ PET uptake, which were more pronounced in women than in men.

**Conclusions:**

Our results suggest that tailoring core AD CSF biomarkers by sex is not necessary for detecting Aβ PET positivity in CU individuals. However, sex differences in their association with Aβ deposition could influence their prognostic or monitoring applications.

**Supplementary Information:**

The online version contains supplementary material available at 10.1186/s13195-025-01844-1.

## Background

The preclinical stage of Alzheimer’s disease (AD) is an optimal window for intervention, with the potential to delay or even prevent the progression to dementia [1, 2]. Biomarkers are crucial to identify this stage, and they also have a role in enriching preventive clinical trials with individuals at higher risk of progression. Core AD biomarkers include amyloid-beta (Aβ) and tau proteins measured in CSF or PET imaging. However, multiple other biomarkers can be measured in cerebrospinal fluid (CSF), including neuronal injury, synaptic, glial and vascular proteins, which can provide valuable insights into the disease’s pathogenesis in this early stage [3–5].

Sex differences have been reported in AD. Two out of three AD patients are women, with postmenopausal women contributing to over 60% of all those affected [6]. Women have a higher resilience to AD pathology in the early stages of the disease [7, 8], and experience a higher pathological burden and steeper decline at later stages [9, 10]. However, our understanding of how sex influences CSF biomarkers remains limited. AD biomarker studies usually correct their analyses by sex, hence treating sex as a confounding variable; as a consequence, sex has generally been neglected as a subject of study when analyzing either clinical or biological features of the disease or the effect of interventions [11, 12].

Most studies that reported the effect of sex did not find differences in the concentrations of CSF Aβ42, Aβ40 or in the CSF Aβ42/40 ratio along the AD continuum [13–16], although one study reported lower levels of CSF Aβ42 in men [17]. In contrast, sex differences are more evident in relation to p-tau, with women showing greater CSF p-tau181 levels and subsequent decline for a given CSF Aβ42 level [14, 18, 19]. Other studies have found differences particularly when the *APOE-*ε4 status is considered, such that women *APOE*-ε4 carriers show higher CSF p-tau181 and t-tau [14, 15, 17, 20, 21]. Furthermore, sex differences in CSF p-tau181 have also been found to differ by both *APOE-*ε4 and disease stage, with differences being more evident in early disease stages in *APOE-*ε4 carriers, and in advanced stages in non-carriers [22].

CSF neurofilament light (NfL) is widely reported to be higher in men compared to women and, in fact, the need of sex-specific reference intervals for CSF NfL has been suggested [4, 23–28]. Yet, evidence on sex differences in other AD-related CSF biomarkers is scarcer and more inconsistent. Interestingly, CSF neurogranin was found to be higher in women than in men in various study populations [4, 26, 27, 29, 30], however, others did not find sex differences [31–33]. CSF IL-6 and MCP-1, along with CSF biomarkers related to vascular dysfunction such as CSF sVCAM-1 and sICAM-1 have been reported to be higher in men [16, 34, 35]. Finally, evidence of sex differences in CSF glial and vascular biomarkers is also conflicting. CSF YKL-40, along with several complement proteins, were found to be higher in men across the AD clinical spectrum and also in the preclinical stage [4, 36], while other studies did not find differences in CSF YKL-40 [35] or sTREM2 [37]. Notably, prior studies have shown that the relationship between AD pathology and neurodegeneration-related gliosis as measured with CSF biomarkers may be modified by sex [38, 39]. A recent study found that women with higher Aβ burden on PET showed stronger associations of CSF YKL-40 and GFAP with CSF p-tau181 than men, while in men with greater tau burden, CSF YKL-40 and hippocampal volume were more strongly associated than in women [39].

Overall, the potential effect of sex in AD CSF biomarkers has been generally underexplored, particularly in cognitively unimpaired individuals (CU), and the available results are inconsistent. Moreover, whether there is a sex effect not only in CSF biomarker levels but on their diagnostic and prognostic capacity, particularly important in early disease stages, remains unclear. Studying the effect of sex in AD-related CSF biomarkers will improve our understanding of the observed heterogeneity in AD pathogenesis, as well as inform about the accurate use and interpretation of biomarkers in AD prevention trials. Therefore, the main aim of this study was to investigate whether there are sex differences in AD CSF biomarkers, their performance to detect Aβ PET positivity, and their association with Aβ pathology in CU individuals. We investigated several CSF biomarkers, which reflect multiple pathophysiological mechanisms relevant in AD, in two independent cohorts of CU individuals. We performed sex-stratified analyses and tested interactions with sex to characterize its effect on CSF biomarker levels and their associations with Aβ pathology on PET cross-sectionally and longitudinally.

## Methods

### Study cohorts

The ALFA + cohort is a nested longitudinal study from the ALFA (for ALzheimer’s and FAmilies) study [40], in which late middle aged (between 45 and 65 years old at baseline [*i.e.* at their inclusion in ALFA]) CU participants were invited to participate based on their specific AD risk profile and were comprehensively characterised, including CSF, magnetic resonance imaging (MRI), and positron emission tomography (PET) biomarker acquisition. The current study included data from 400 ALFA + participants with baseline CSF biomarkers. Among them, 340 also had an Aβ PET at baseline, and 204 had available Aβ PET at the follow-up visit (average follow-up of 3.25 years, range: 1.30 to 5.85 years). In ALFA +, 52% of Aβ PET visits occurred within 3 months of a lumbar punction procedure and 97% occurred within a year of lumbar punction procedure.

The Wisconsin Registry for Alzheimer's Prevention (WRAP) and Wisconsin Alzheimer's Disease Research Center (WADRC) at the University of Wisconsin (Wisconsin, USA) were included as a validation/replication cohort. WRAP is comprised of initially CU, middle-aged (between 40 and 65 years old) adults enriched for parental history of dementia presumed due to AD [41] followed-up every two years. At each visit, the participants undergo comprehensive medical and cognitive evaluations. The WADRC clinical core is comprised of participants across the AD spectrum who undergo cognitive testing and physical exams annually or biennially [42]. The present study included data from 548 WRAP/WADRC CU participants with available CSF biomarkers. 183 participants also had an Aβ PET imaging visit within 2 years of CSF sample collection and 143 had follow-up Aβ PET imaging data (average follow-up of 3.17 years, range: 1.78 to 12.7 years). In WRAP/WADRC, 48% of PET visits occurred on the same day as the lumbar puncture procedure and 75% occurred within 30 days of the lumbar puncture procedure. In this cohort, we selected the CSF sample and Aβ PET imaging that took place closest in time (within a maximum of two years) for cross-sectional analyses of CSF biomarker and Aβ PET Centiloid values. For analyses of change in Centiloid values, baseline CSF was defined as CSF closest to the participant’s first Aβ PET imaging visit (within a maximum of two years).

In the two cohorts, sex was categorized based on self-report. Global cognitive performance was assessed with the Mini Mental State Examination (MMSE) [43] and the Preclinical Alzheimer Cognitive Composite (PACC) [44, 45].

Available baseline CSF biomarkers in both cohorts included core AD biomarkers (Aβ42, Aβ42/40, p-tau181, p-tau181/Aβ42), as well as neurodegeneration (t-tau, NfL), synaptic dysfunction (neurogranin, α-synuclein), glial reactivity (sTREM2, YKL-40, GFAP, S100B) and neuroinflammation (IL-6) biomarkers. In ALFA +, additional CSF biomarkers were available, including biomarkers for tau pathology (p-tau217, p-tau231), synaptic dysfunction (SNAP25, GAP-43, synaptotagmin-1), vascular dysregulation (sICAM-1, sVCAM-1), and neuroinflammation (MCP-1).

### CSF sample acquisition, processing, and biomarker measurements

CSF procedures in the ALFA + and the WRAP/WADRC cohorts have been reported previously [4, 46]. All measurements, except for CSF p-tau217, were performed at the Clinical Neurochemistry Laboratory, Sahlgrenska University Hospital, Mölndal, Sweden.

In both cohorts, CSF t-tau and p-tau181 were measured using the electrochemiluminescence immunoassays Elecsys® Total-tau CSF and phospho-tau(181P) CSF on a fully automated cobas e601 instrument (Roche Diagnostics International Ltd.). CSF Aβ40, Aβ42, NfL, neurogranin, α-synuclein, sTREM2, YKL-40, GFAP, S100B and IL-6 were measured with the prototype NeuroToolKit (Roche Diagnostics International Ltd.) on a cobas e411 or e601 instrument as previously described in both cohorts [4, 47]. CSF Aβ42 measured with the Elecsys® β-Amyloid (1–42) CSF immunoassay was additionally available in ALFA +, and it was used as an individual biomarker as well as combined with CSF p-tau181 as the p-tau181/Aβ42 ratio.

In ALFA +, measurement of CSF p-tau231 was performed using a research ELISA assay using cis-conformational selective monoclonal antibody (ADx253, ADx NeuroSciences), which was described previously [48]. Eli Lilly and Company provided the measurements of the previously published in-house assay for CSF p-tau217 [49] using the Meso Scale Discovery platform (MSD). CSF SNAP25 and synaptotagmin-1 concentrations were measured by immunoprecipitation mass spectrometry following a previously established protocol [50]. In particular, the longer soluble forms of SNAP25 including at least amino acids 32 through 40 (SNAP 25aa40) were evaluated herein. CSF GAP-43 was measured by ELISA as previously described [51].

CSF sICAM-1 and sVCAM-1 were measured with the MSD Vascular Injury (human) Panel 2 kit #K15198D. MCP-1 was measured with the MSD V-Plex Chemokine (human) Panel 1 kit #K15047D.

### Aβ PET acquisition, quantification, and Aβ status determination

In ALFA +, participants underwent [^18^F]flutemetamol Aβ PET scans after a cranial CT scan for attenuation correction on a Biograph mCT scanner (Siemens Healthcare, Erlangen, Germany) at Hospital Clínic, Barcelona, Spain. Participants received an IV bolus dose of 185 MBq (range 104.25–218.3 MBq, mean ± SD 191.75 ± 14.04 MBq), and 90 min after injection, PET data were acquired for 20 min (4 frames of 5 min each, mean ± SD 90.15 ± 7.36 min). PET images were reconstructed in 4 frames of 5 min using the 3-dimensional Ordered Subset Expectation Maximization algorithm by incorporating time of flight and point spread function modeling. Centiloid values were calculated from the mean values of the standard Centiloid target region (http://www.gaain.org/centiloid-project) using the transformation previously calibrated [52]. A nuclear medicine physician visually rated the scans as Aβ-positive or Aβ-negative using standard clinical criteria as specified in the Summary of Product Characteristics of the tracer (https://www.ema.europa.eu/en/documents/product-information/vizamyl-eparproduct-information_en.pdf).

Aβ PET acquisition and quantification in the WRAP/WADRC has been previously described [53]. Participants underwent [^11^C]PiB PET scans on a Siemens EXACT HR + scanner (Siemens Healthcare, Erlangen, Germany). Participants received an IV bolus of 555 [^11^C]PiB (mean ± SD 566.1 ± 33.3 MBq), at the start of a 70-min dynamic [^11^C]PiB PET acquisition. The PET data were reconstructed using a filtered back-projection algorithm (Direct inverse Fourier Transformation; DIFT) with sinogram trimming to a voxel size of 2.57 mm × 2.57 mm × 2.43 mm and matrix dimension of 128 × 128 × 63 and corrected for random events, attenuation of annihilation radiation, dead time, scanner normalization, and scatter radiation using the ECAT v7.2.2 software with segmented attenuation correction. The reconstructed time series were corrected for subject motion and a denoising algorithm was applied to the voxel-based time series. SUVR values were converted to Centiloids following Betthauser et al. [54]. Briefly, WRAP data were downloaded from the GAAIN website (https://www.gaain.org/centiloid-project) and processed locally to define the relationship between SUVR and Centiloids [55]. Visual rating of Aβ-positive or Aβ-negative status was achieved on the native space DVR images that were all scaled uniformly from 0.0 to 2.5, and displayed using a color map (the ACTC activation color map) that provided distinct shades of color for demarcating PiB positivity (which corresponds to a Centiloid of approximately 18 [56]).

### Statistical analyses

We assessed the normality of CSF biomarker distributions using the Kolmogorov–Smirnov test and visual inspection of histograms. In ALFA +, all CSF biomarkers except for CSF Aβ42, the Aβ42/40 ratio, sTREM2 and MCP-1 were log10-transformed to approximate normality. In WRAP/WADRC, all CSF biomarkers were log10-transformed, except for CSF Aβ42 and the CSF Aβ42/40 and p-tau181/Aβ42 ratios.

Differences in age or education between sexes were evaluated using t-test, while differences in prevalence of *APOE-*ε4 or Aβ positivity were evaluated with Chi-squared (χ2) test. Cognitive performance or Aβ burden (Aβ PET Centiloid values) were compared between sexes using one-way analysis of covariance (ANCOVA) adjusting for age and *APOE-*ε4 status. Models testing differences in cognitive measures were further adjusted by years of education.

Differences in CSF biomarkers between men and women were tested in three different models: (1) unadjusted one-way analysis of variance (ANOVA), (2) with an ANCOVA adjusting for the effect of age, and (3) with an ANCOVA adjusting for both the effects of age and *APOE-*ε4 status. Additional models included the interaction term between sex and Aβ status as determined by CSF Aβ42/40. CSF Aβ42/40 status was defined using previously established in-house positivity thresholds for each cohort. In ALFA, the threshold (Aβ42/40 < 0.071) was derived using Gaussian Mixture Modelling [4], and in WRAP/WADRC, the threshold (Aβ42/40 < 0.046) was determined based on the Youden's index [47].

We performed sex-stratified receiver-operating characteristic (ROC) analyses to evaluate core AD CSF biomarkers’ discrimination accuracy to detect Aβ PET-positive participants, as defined by visual reads. We calculated the biomarker’s sensitivity, specificity and the area under the curve (AUC) values with their 95% confidence intervals (CI) in women and men, and the AUCs were compared using DeLong’s tests.

Finally, we used linear regression models to study the associations between baseline CSF biomarkers, and both baseline and follow-up Aβ burden as measured with Aβ PET Centiloid values. Change in Aβ PET Centiloid values at follow-up was calculated subtracting the values at follow-up visit from the baseline values.

We ran independent linear regression models with Centiloid values (at baseline or its change) as the outcome, adjusting by age and *APOE*-ε4 status. We tested the modifying effect of sex by including the interaction term between sex and each CSF biomarker in these models. Additionally, we ran sex-stratified models to evaluate the sex-specific main effects of each baseline CSF biomarker on Aβ PET uptake. All models assessing effects of sex on Centiloid change included the time difference between the baseline and the follow-up visit as a covariate. Furthermore, given the non-linear association of Centiloid change with respect to baseline Centiloid values, we performed a sensitivity analysis by restricting models including Centiloid change to baseline Centiloid values below 30.

For all analyses, we applied a false discovery rate (FDR) correction for multiple comparisons across all biomarkers tested, following the Benjamini-Hochberg [57] procedure. All tests were 2-tailed, with a significance level of α = 0.05. Statistical analyses and figures were performed using R (version 4.2.2).

## Results

### Participants’ characteristics

ALFA+ included 400 CU individuals (mean baseline MMSE = 29.1, mean baseline PACC = 0.01) and WRAP/WADRC included 548 CU individuals (mean baseline MMSE = 29.3, mean baseline PACC = −0.08). Both cohorts had a higher representation of women than men (61.5% in ALFA *vs.* 67.3% in WRAP/WADRC), and age range at baseline was also similar between the two cohorts (mean age of 61.1 years ALFA+; mean age of 61.4 years in WRAP/WADRC).

The frequency of *APOE*-ε4 carriers was higher in ALFA + (54.0%) than in WRAP/WADRC (36.6%). The percentage of participants who were CSF Aβ-positive was also higher in ALFA+ (33.8% *vs.* 17.2% in WRAP/WADRC. Nevertheless, the frequency of participants with a positive Aβ PET visual read was 12.4% in ALFA+ and 17.5% in WRAP/WADRC.

Demographics and baseline characteristics by sex in both cohorts included in the study are summarized in Table 1**.** There were no statistically significant age differences between men and women in either of the two cohorts. In both cohorts, men had higher years of education compared to women. Of note, in ALFA+, the frequency of *APOE-ε*4 carriers was higher in men than in women (61.7% in men *vs. *49.2% in women; *P* = 0.02). Despite this, there were no sex differences in Aβ burden on Aβ PET or frequency of Aβ-positive individuals as measured with CSF nor with Aβ PET in either cohort.

**Table 1:** Demographics and baseline characteristics of the study cohorts by sex

	ALFA+				WRAP/ADRC			
	**All**	**Women**	**Men**	***P*-value**	**All**	**Women**	**Men**	***P-*value**
	**N = 400**	**N = 246**	**N = 154**		**N = 548**	**N = 369**	**N = 179**	
**Age, years**	61.1 (4.73)	60.8 (4.85)	61.7 (4.51)	0.06	61.4 (7.6)	61.0 (7.53)	62.3 (7.83)	0.05
***APOE-ε*4 carriers, n (%)**	216 (54.0)	121 (49.2)	95 (61.7)	**0.02**	194 (36.6)	133 (37.7)	61 (35.4)	0.69
**Education, years**	13.5 (3.55)	13.0 (3.61)	14.4 (3.30)	** < 0.001**	16.2 (2.46)	15.9 (2.28)	16.8 (2.66)	** < 0.001**
Menopause status* (post-menopause), n (%)	-	174 (95.6)	-	**-**	-	106 (91.4)	-	**-**
**MMSE**	29.1 (0.94)	29.1 (0.97)	29.3 (0.88)	**0.01**	29.3 (0.94)	29.4 (0.90)	29.1 (0.99)	**0.01**
**PACC**	0.01 (0.69)	−0.02 (0.71)	0.03 (0.67)	0.40	−0.08 (1.04)	0.14 (0.95)	−0.53 (1.07)	** < 0.001**
**Aβ PET Centiloid†**	2.81 (16.8)	2.88 (18.1)	2.70 (14.3)	0.33	14.1 (26.4)	15.2 (28.8)	11.2 (20.1)	0.30
**Aβ PET-positive†, n (%)**	42 (12.4)	26 (12.3)	16 (12.5)	0.99	32 (17.5)	23 (19.7)	9 (16.7)	0.79
**CSF Aβ-positive, n (%)**	135 (33.8)	81 (32.9)	54 (35.1)	0.74	82 (17.2)	51 (16.1)	31 (19.8)	0.36

Data are expressed as mean (M) and standard deviation (SD) or percentage (%), as appropriate. T-test was used to compare age and education and Pearson’s χ2 test to compare prevalence of *APOE*-ε4 status and Aβ PET or CSF Aβ positivity between men and women. MMSE and PACC scores and Centiloid values were compared with ANCOVA adjusted by age and *APOE*-ε4 status. Models testing MMSE and PACC scores were further adjusted by years of education. Significant *P*-values are shown in bold

Abbreviations: Aβ, amyloid-β; *APOE*, Apolipoprotein E; CSF, cerebrospinal fluid; MMSE, Mini-Mental State Examination; PACC, Preclinical Alzheimer’s Cognitive Composite; PET, Positron Emission Tomography

^*^Information on menopause status was available for 182 women in ALFA+ and for 116 women in WRAP/ADRC. †Aβ PET was available for n = 127 men and n = 213 women in ALFA+, and n = 59 men and n = 124 women in WRAP/WADRC. In WRAP/WADRC, *APOE* genotype was missing for 5 men and 16 women

In relation to sex differences in cognitive performance at baseline, after adjusting by age, *APOE*-ε4 status and years of education, men had higher baseline MMSE scores compared to women in ALFA+ (29.3 vs 29.1, respectively; *P* = 0.01), but no differences were found in PACC scores. In contrast, in WRAP/WADRC, women outperformed men at both MMSE (29.1 vs 29.4 in men and women, respectively; *P* = 0.01) and PACC (−0.53 and 0.14 in men and women, respectively, *P* < 0.001).

### Sex differences in CSF biomarker levels

Table 2 summarizes the results on sex differences in baseline CSF biomarker levels in ALFA+ and WRAP/WADRC cohorts.

**Table 2:** Baseline CSF biomarkers by sex

	**ALFA+ **				**WRAP/WADRC**			
**CSF biomarkers**	**All (N = 400)**	**Women (N = 246) **	**Men (N = 154)**	**Cohen’s *d***	**All** **(N = 548)**	**Women (N = 369)**	**Men (N = 179)**	**Cohen’s *d***
**Core AD CSF biomarkers**								
**A**β**42 (pg/ml)**	1085 (323)	1124 (322)	1023 (316)	0.32^*†^	938 (382)	941 (391)	936 (368)	0.01
**A**β**42/40**	0.08 (0.02)	0.08 (0.02)	0.07 (0.02)	0.07	0.07 (0.02)	0.07 (0.02)	0.06 (0.02)	0.05
**p-tau181/A**β**42**	0.01 (0.01)	0.02 (0.01)	0.02 (0.01)	−0.10	0.02 (0.02)	0.02 (0.02)	0.02 (0.01)	0.02
**p-tau181 (pg/ml)**	16.3 (7.57)	16.7 (8.22)	15.6 (6.35)	0.15	17.3 (6.82)	17.3 (6.90)	17.4 (6.65)	−0.02
**p-tau217 (pg/ml)**	7.66 (6.10)	8.05 (6.71)	7.05 (4.94)	**0.17**^**† ‡**^	-	-	-	-
**p-tau231 (pg/ml)**	8.41 (6.78)	8.68 (7.44)	7.97 (5.58)	0.10	-	-	-	-
**Non-AD-specific CSF biomarkers**								
**t-tau (pg/ml)**	198 (73.1)	202 (77.0)	191 (65.9)	0.16	198 (72.5)	197 (72.5)	200 (72.6)	−0.05
**NfL (pg/ml)**	83.8 (36.0)	78.7 (37.1)	92.1 (32.4)	−0.37^*† ‡^	90.5 (57.8)	82.7 (40.7)	101.9 (49.7)	−0.44^*† ‡^
**neurogranin (pg/ml)**	800 (331)	836 (346)	743 (297)	0.30^*† ‡^	784 (319)	806 (325)	742 (305)	0.19^*† ‡^
**GAP-43 (pg/ml)**	2844 (1198)	2921 (1251)	2722 (1101)	0.17	-	-	-	-
**SNAP25 (pM)**	21.7 (3.15)	21.8 (3.27)	21.5 (2.96)	0.08	-	-	-	-
**synaptotagmin-1 (pM)**	52.3 (14.0)	53.6 (14.8)	50.2 (12.2)	0.24^*† ‡^	-	-	-	-
**α-synuclein (pg/ml)**	234 (254)	252 (308)	206 (125)	0.18^*^	159 (68.1)	159 (68.8)	161 (66.8)	−0.04
**GFAP (ng/ml)**	7.72 (2.64)	7.49 (2.64)	8.09 (2.60)	−0.23^*† ‡^	9.01 (3.28)	8.78 (3.26)	9.49 (3.29)	−0.22^*† ‡^
**S100B (ng/ml)**	1.02 (2.36)	1.00 (2.18)	1.06 (2.60)	−0.23^*^	1.15 (0.30)	1.11 (2.50)	1.23 (0.37)	−0.39^*† ‡^
**sTREM2 (ng/ml)**	7.96 (2.23)	7.95 (2.22)	7.96 (2.33)	−0.01	7.91 (2.43)	7.87 (2.45)	8.00 (3.4)	−0.07
**YKL-40 (ng/ml)**	148 (54.1)	150 (54.5)	144 (53.4)	0.10	143 (53.0)	141 (53.7)	149 (51.2)	−0.15
**IL-6 (pg/ml)**	4.04 (2.01)	3.98 (2.16)	4.15 (1.74)	−0.07	4.59 (3.04)	4.39 (2.84)	5.00 (3.4)	**−0.19**^**† ‡**^
**MCP-1 (pg/ml)**	391 (99.3)	383 (99.0)	403 (99.0)	−0.21	-	-	-	-
**sICAM-1 (pg/ml)**	2407 (683)	2328 (669)	2531 (689)	−0.30^*† ‡^	-	-	-	-
**sVCAM-1 (pg/ml)**	6623 (2007)	6319 (1962)	7096 (1990)	−0.39^*† ‡^	-	-	-	-

Data are expressed as mean (M) and standard deviation (SD). Comparisons of CSF biomarkers were performed using three models: 1) unadjusted ANOVA, 2) ANCOVA adjusted for age, and 3) ANCOVA adjusted for age and *APOE*-ε4 status. Cohen's *d* values represent the effect sizes for the unadjusted group differences. Statistically significant effects in any model are shown in bold

^*^P-value < 0.05 in unadjusted model

^†^*P*-value < 0.05 in models adjusted by age

^‡^*P*-value < 0.05 in models adjusted by age and *APOE*-ε4 status

In ALFA, all sex differences were maintained after FDR multiple comparison correction except for GFAP in all models, and for S100B, synaptotagmin-1 and α-synuclein in unadjusted models. In WRAP/WADRC, all sex differences were maintained after FDR multiple comparison correction except for GFAP in the unadjusted model and in the age-adjusted model

Abbreviations: Aβ40, amyloid-β 40; Aβ42, amyloid-β 42; GAP-43; growth-associated protein-43; GFAP, glial fibrillary acidic protein; IL-6, interleukin 6; MCP-1, monocyte chemoattractant protein-1; NfL, neurofilament light; p-tau, phosphorylated tau; sICAM-1, soluble intercellular adhesion molecule-1; SNAP25; synaptosomal-associated protein-25; sTREM2, soluble triggering receptor expressed on myeloid cells 2; t-tau, total tau; sVCAM-1, soluble vascular cell adhesion molecule-1

In ALFA+, unadjusted models showed that CSF Aβ42 was lower in men (Cohen’s *d* = 0.32; *P* < 0.05), while the neurodegeneration biomarker CSF NfL (Cohen’s *d* = −0.37; *P* < 0.05), and the vascular biomarkers CSF sICAM-1 and sVCAM-1 (Cohen’s *d* = −0.30 and Cohen’s *d* = −0.39, respectively; *P* < 0.05) were higher in men. In contrast, the synaptic biomarker CSF neurogranin was higher in women (Cohen’s *d* = 0.30; *P* < 0.05). Men also had higher levels of the astrocyte reactivity biomarkers CSF GFAP and S100B (Cohen’s *d* = −0.23; *P* < 0.05, for both), and lower levels of synaptic biomarkers CSF synaptotagmin-1 and α-synuclein (Cohen’s *d* = 0.24 and Cohen’s *d* = 0.18, respectively; *P* < 0.05), although these differences did not survive multiple comparison correction. When adjusting for the effect of age, the sex differences in CSF Aβ42, NfL, neurogranin, synaptotagmin-1, GFAP, sICAM-1 and sVCAM-1 were maintained. In addition, CSF p-tau217 was found significantly higher in women (Cohen’s *d* = 0.17; *P* < 0.05). When further adjusting for the effect of *APOE-*ε4 status, all differences were maintained except for CSF Aβ42 (Cohen’s *d* =0.22; *P* = 0.06).

In the WRAP/WADRC cohort, the sex differences in CSF NfL, neurogranin, GFAP, and S100B were replicated, also when models were adjusted by age or by age and *APOE-*ε4 status (Table 2). In contrast, the difference in CSF Aβ42 was not replicated in WRAP/WADRC cohort, while CSF IL-6 was significantly higher in men in this cohort (Cohen’s *d* = −0.19; *P* <0.05). CSF p-tau217, synaptotagmin-1, MCP-1, sVCAM-1 and sICAM-1 were not available in WRAP/WADRC.

Of note, in ALFA+ and WRAP/WADRC cohorts, there were no significant interactions between sex and CSF Aβ status on baseline CSF biomarker levels (Supplementary Table 1 in the Additional file 1), suggesting the observed differences exist regardless of CSF Aβ levels.

### Sex-specific performance of core AD CSF biomarkers in detecting Aβ PET positivity

We next investigated whether the performance of core AD CSF biomarkers to detect Aβ pathology, as assessed by Aβ PET, in CU individuals differed by sex. To that aim, we run sex-specific ROC analyses to evaluate their performance in identifying participants with a positive Aβ PET visual read. Detailed diagnostic parameters by sex in the two study cohorts are shown in Table 3.

**Table 3:** Sex-specific prediction of Aβ PET positivity using core AD CSF biomarkers

	**ALFA+ **											
	**Women**						**Men**					***P*-value**
	**AUC [95% CI]**	**Specificity**		**Sensitivity**		**Youden's index**	**AUC [95% CI]**	**Specificity**	**Sensitivity**	**Youden's index**		
**Aβ42**	76.3 [66.4–86.3]	62.3		80.0		142	70.6 [57.3–83.9]	35.9	100	136		0.50
**Aβ42/40**	91.0 [83.8–98.3]	83.3		92.3		176	83.8 [72.3–95.3]	75.9	93.8	170		0.35
**p-tau181/Aβ42**	93.0 [86.2–99.8]	89.3		92.0		181	88.4 [80.7–96.1]	66.7	100	167		0.38
**p-tau181**	84.1 [75.4–92.8]	84.4		76.9		161	77.3 [66.4–88.3]	58.7	87.5	146		0.34
**p-tau217**	93.0 [87.7–98.3]	74.2		80.8		155	87.1 [77.7–96.6]	71.4	87.5	159		0.29
**p-tau231**	92.2 [85.1–99.3]	85.0		73.1		158	85.2 [74.3–96.1]	76.6	81.3	158		0.29
	**WRAP/WADRC**											
	**Women**						**Men**					***P*-value**
	**AUC [95% CI]**	**Specificity**	**Sensitivity**		**Youden's index**		**AUC [95% CI]**	**Specificity**	**Sensitivity**		**Youden's index**	
**Aβ42**	87.3 [80.1–94.6]	74.7	92.3		167		79.3 [64.7–93.7]	71.4	75.0		146	0.29
**Aβ42/40**	96.2 [92.4–100]	87.9	96.2		184		93.0 [86.5–99.5]	85.7	91.7		177	0.13
**p-tau181/Aβ42**	96.3 [92.3–100]	87.9	96.1		184		93.7 [86.1–100]	83.7	91.7		175	0.31
**p-tau181**	81.4 [71.7–91.2]	70.1	80.8		152		73.3 [57.7–88.9]	40.8	100		141	0.44

Sex-specific ROC analyses for the discrimination between Aβ PET-positive and Aβ PET-negative individuals, as defined by Aβ PET visual reads. AUC differences between men and women were tested using a two-sided DeLong’s test and nominal *P-*values are shown

Abbreviations: Aβ40, amyloid-β 40; Aβ42, amyloid-β 42; AUC, area under the curve; CI, confidence interval; p-tau, phosphorylated tau;

In the ALFA+ cohort, the best performing biomarkers at identifying Aβ PET-positive participants were CSF p-tau217, the ratio between p-tau181 and Aβ42 (p-tau181/Aβ42) and p-tau231 in both women and men. CSF p-tau217 and the p-tau181/Aβ42 ratio showed the highest AUCs in women (AUC of 93.0 for both) and were followed by CSF p-tau231 (AUC of 92.2) and Aβ42/40 (AUC of 91.0). Similar results were found in men, with the CSF p-tau181/Aβ42 ratio reaching an AUC of 88.4, closely followed by CSF p-tau217 (AUC of 87.1), p-tau231 (AUC of 85.2) and Aβ42/40 (AUC of 83.8). Although the classification performances were generally higher in women than in men, DeLong's tests showed no statistically significant differences in AUCs of any biomarker between sexes **(**all *P*-values > 0.05; Table 3**).**

Similarly, in the WRAP/WADRC cohort, CSF p-tau181/Aβ42 and Aβ42/Aβ40 had the best performance in both sexes, reaching AUCs of 96.3 and 96.2, respectively, in women, and AUCs of 93.7 and 93.0, respectively, in men, with no statistically significant differences between sexes (all *P*-values > 0.05; Table 3). CSF p-tau217 and p-tau231 were not available in WRAP/WADRC.

### Association of CSF biomarkers with Aβ pathology as measured by PET

We analysed whether sex modified the association between baseline CSF biomarkers and baseline and longitudinal changes in Aβ PET Centiloid values.

**Fig. 1:**
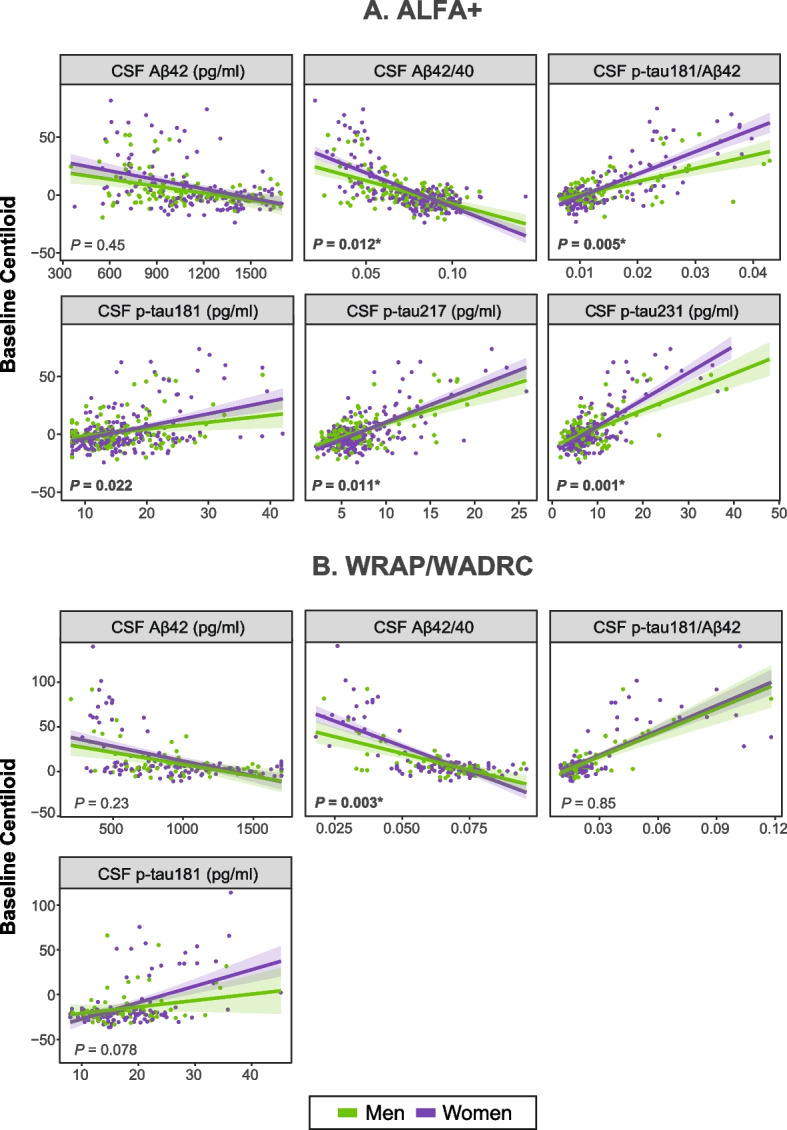
Scatter plots showing the associations between baseline core AD CSF biomarkers and Aβ PET Centiloid values, stratified by sex. Each point represents an individual's CSF biomarker value. Nominal *P*-values refer to the interaction term between sex and each CSF biomarker, adjusted for age and *APOE-*ε4 status. Significant *P*-values are highlighted in bold. *P*-values marked with an asterisk (*) remain significant after false discovery rate (FDR) correction for multiple comparisons

**Fig. 2:**
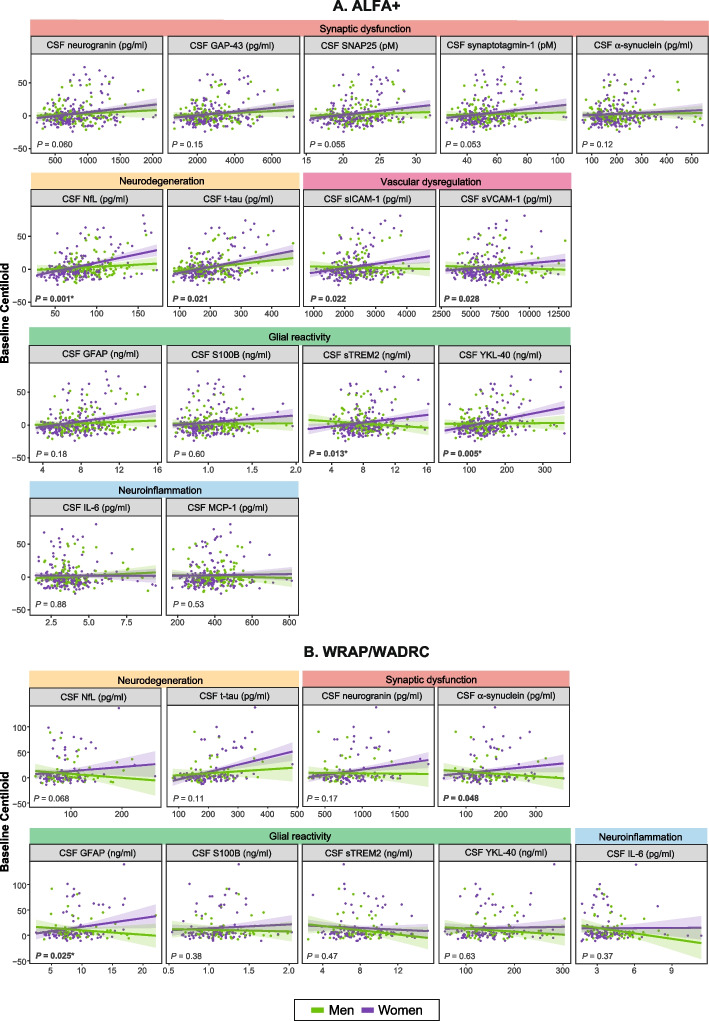
Scatter plots showing the associations between baseline non-AD-specific CSF biomarkers and Aβ PET Centiloid values, stratified by sex. Each point represents an individual's CSF biomarker value. Nominal *P*-values refer to the interaction term between sex and each CSF biomarker, adjusted for age and *APOE-*ε4 status. Significant *P*-values are highlighted in bold. *P*-values marked with an asterisk (*) remain significant after false discovery rate (FDR) correction for multiple comparisons

In ALFA+, sex modified the association between baseline core AD biomarkers and baseline Aβ PET Centiloid values (Supplementary Table 2 in the Additional file 1). Specifically, as shown in sex-stratified analyses (Supplementary Table 3 in the Additional file 1), the associations of lower CSF Aβ42/40, and higher CSF p-tau181/Aβ42, p-tau231 and p-tau217 with higher Aβ PET Centiloid values were greater in women than in men (CSF Aβ42/40 x sex interaction: β= 0.11, *P* = 0.012; CSF p-tau181/Aβ42 x sex interaction: β= −0.29, *P=*0.005; CSF p-tau231 x sex interaction: β= −0.14, *P*=0.001; and CSF p-tau217 x sex interaction: β= −0.11, *P*=0.011; Fig. 1, Supplementary Table 2 in the Additional file 1). In addition, higher baseline CSF NfL and glial reactivity biomarkers CSF YKL-40 and sTREM2 were associated with higher Aβ PET Centiloid values specifically in women (CSF NfL x sex interaction: β= −0.18, *P* = 0.001; CSF YKL-40 x sex interaction: β= −0.15, *P* = 0.005; and CSF sTREM2 x sex interaction: β= −0.14, *P* = 0.013; Fig. 2, Supplementary Table 2 in the Additional file 1). Significant interactions in the same direction were also found for CSF p-tau181, t-tau, sICAM-1 and sVCAM-1, but they did not survive multiple comparison correction. Notably, all synaptic biomarkers were significantly associated with Centiloid values in women but not in men in stratified analyses (Supplementary Table 3 in the Additional file 1). The interaction terms, particularly for CSF neurogranin, SNAP25, and synaptotagmin-1, were near the threshold of statistical significance (*p* ≈ 0.05–0.06) (Fig. 2, Supplementary Table 2 in the Additional File 1).

 The stronger association of CSF Aβ42/40 with Aβ PET Centiloid values in women compared to men was replicated in the WRAP/WADRC cohort (CSF Aβ42/40 x sex interaction: β= 0.35, *P* = 0.003), and a non-significant trend in the same direction was found for CSF p-tau181 (CSF p-tau181 x sex interaction: β= −0.28, *P* = 0.078; Fig. 1, Supplementary Table 2 in the Additional file 1). In this cohort, sex also modified the cross-sectional associations of α-synuclein and GFAP with Aβ PET Centiloid values, these being stronger in women compared to men (CSF α-synuclein x sex interaction: β= −0.32, *P* = 0.048; and CSF GFAP x sex interaction: β= −0.36, *P* = 0.025; Fig. 2, Supplementary Table 2 in the Additional file 1). The interactions between sex and CSF Aβ42/40 and GFAP survived multiple comparison correction.

Finally, we tested whether sex modified the associations of baseline CSF biomarkers and longitudinal change in Aβ PET uptake. In ALFA+, a significant modifying effect of sex was found on the association between lower baseline CSF Aβ42/40 and increased Aβ PET uptake at follow-up (CSF Aβ42/40 x sex interaction: β=0.15, *P* = 0.008). Similarly, sex significantly modified the associations between higher baseline CSF p-tau181/Aβ42, p-tau217, p-tau231, and NfL with increased Aβ PET uptake at follow-up (CSF p-tau181/Aβ42 x sex interaction: β= −0.26, *P* = 0.006; CSF p-tau217 x sex interaction: β= −0.13, *P* = 0.033; CSF p-tau231 x sex interaction: β= −0.13, *P* = 0.035; and CSF NfL x sex interaction: β= −0.16 *P* = 0.031). Sex-stratified analyses showed stronger associations of all these biomarkers with longitudinal Aβ PET among women. However, the interaction analyses results did not survive multiple comparisons correction (Supplementary Figs. 1 and 2 in the Additional file 1; Supplementary Tables 4 and 5 in the Additional file 1).

In the WRAP/WADRC cohort we did not find any significant modifying effect of sex in the associations between baseline CSF biomarkers and longitudinal change in Aβ PET Centiloid values (Supplementary Figs. 1 and 2 in the Additional file 1; Supplementary Tables 4 and 5 in the Additional file 1).

For both ALFA+ and WRAP/WADRC, sensitivity analyses restricted to baseline Centiloid values below 30 yielded results similar to those observed in the full cohort (Supplementary Table 6 in the Additional file 1).

## Discussion

In this study, we investigated sex differences in multiple AD-related CSF biomarkers, their performance at identifying Aβ PET positivity, and their association with Aβ pathology measured by PET in two well characterized cohorts of CU individuals. Our main findings are: 1) In both cohorts, we observed that the synaptic biomarker CSF neurogranin is higher in women, while the neurodegeneration marker CSF NfL and astrocytic markers CSF GFAP and S100B are increased in men; 2) The discrimination accuracy of core AD CSF biomarkers to identify CU Aβ PET-positive participants did not differ by sex; 3) Higher baseline levels of several CSF biomarkers, including core AD biomarkers, NfL and glial biomarkers, showed a stronger association with higher Aβ PET uptake in women than in men.

In line with previous studies [13–16], we did not find sex differences in CSF Aβ42/40 ratio in either of the two cohorts included in this study. Although men had lower levels of CSF Aβ42 in ALFA+, this difference was not significant after accounting for *APOE-*ε4, suggesting that it was probably explained by the higher frequency of *APOE-*ε4 carriership in men than in women in this cohort. Importantly, CSF Aβ42/40, in contrast to CSF Aβ42, partly accounts for interindividual differences in Aβ production and is a more accurate biomarker of Aβ pathology [58]. Based on previous evidence supporting a higher vulnerability to tau pathology in women [14, 15, 18–21], we hypothesized that tau CSF biomarkers would be elevated in women. In our study, only the sex difference in p-tau217, available in ALFA+, reached statistical significance. This result is in line with women showing a higher degree of downstream tau pathology already in preclinical AD, which would possibly become more evident in subsequent disease stages. However, it is important to note that p-tau levels in biofluids rise earlier than tau deposition detected by Tau PET imaging. As such, they are more closely associated with biomarkers of Aβ pathology and, accordingly to the new Alzheimer’ Association criteria [59], are classified as phosphorylated and secreted AD tau (T_1_) biomarkers. This may help explain the lack of robust sex differences in CSF p-tau levels observed in our study [60–63]. Of note, age at menopause or hormone therapy use have been suggested as modifying factors for increased tau vulnerability [64]. Interestingly, testosterone levels have also been found to influence tau levels [65]. Both ALFA+ and WRAP/WADRC cohort are comprised of mostly postmenopausal women with low frequency of hormone therapy use, but future studies should examine the potential modifying role of hormonal exposure on CSF tau biomarkers.

Besides the core AD CSF biomarkers, our study provides evidence of sex differences in several CSF biomarkers reflecting Aβ-downstream processes. The sex difference in the neurodegeneration biomarker CSF NfL was robustly found in both cohorts and it is aligned with previous studies [4, 23–28]. It would be plausible to suggest that this difference is indicating a higher degree of neurodegeneration in men. However, other potential explanations could be physiological differences such as a higher axonal turnover or the higher proportion of brain white matter in men [66]. Men also showed elevated biomarkers of astrocyte reactivity (*i.e*. CSF GFAP and S100B) in both cohorts. Vascular dysregulation biomarkers CSF sICAM-1 and sVCAM-1 were available in ALFA+ and were also elevated in men. Higher levels of these biomarkers in men have been reported [16, 34, 35], although here we determined these differences specifically in CU participants. Other inflammatory markers such as CSF IL-6 or MCP-1 had been found elevated in men [35]. In WRAP/WADRC cohort, CSF IL-6 was significantly increased in men, and in ALFA+ a trend towards the same direction was observed for both CSF IL-6 and MCP-1. These results support a higher degree of neuroinflammation and vascular pathology in men than in women, even despite the fact both ALFA+ and WRAP/WADRC cohorts include participants with an overall low vascular risk and low frequency of comorbidities. Further studies are needed to determine whether the observed sex differences in the inflammatory profile persist in older ages or more advanced disease stages and whether they influence disease progression. Higher CSF neurogranin in women was a robust finding in both cohorts, and it is in line with previous literature [26, 27, 29, 30]. In ALFA+, we had measures for additional synaptic biomarkers, namely CSF SNAP25, GAP-43 and synaptotagmin-1, all of them presynaptic proteins. Of those, CSF synaptotagmin-1 was significantly higher in women with respect to men. We had previously reported that these synaptic proteins increase in preclinical AD, and are differentially associated with age or *APOE-*ε4 status [67]. Although we adjusted by these factors in our models, they could still be confounding the effects of sex to some extent.

Overall, we showed that women tend to have higher levels of CSF synaptic biomarkers, while men exhibit higher levels of CSF biomarkers associated with neurodegeneration, astrocyte reactivity, and vascular pathology. Importantly, most of the results were replicated in both cohorts and all observed sex differences, except for CSF Aβ42 in ALFA+, were maintained after adjustment for age and *APOE-*ε4 status. This fact, along with the lack of significant interactions with CSF Aβ status, suggests sex differences in the levels of CSF biomarkers reflecting AD-related processes are, at least partly, independent from the degree of Aβ pathology.

A novelty of our study is that we specifically evaluated whether the capacity of core AD CSF biomarkers to detect Aβ PET positivity in CU individuals differed by sex. Importantly, our results indicate that, despite the differences in some CSF biomarker levels between sexes, there are no significant effects of sex in their discrimination performance. This is a relevant finding given the current use of these core CSF biomarkers in AD prevention trials, as it implies that there is no need to tailor the use of this biomarkers for the identification of CU individuals with detectable Aβ accumulation in the brain and therefore, with preclinical AD.

When we examined the modifying role of sex in the association between CSF biomarkers and Aβ pathology in the brain as measured by PET, in ALFA+ we found that several CSF biomarkers (Aβ42/40, tau, NfL, glial biomarkers and a trend for synaptic biomarkers) had a greater association with higher Centiloid values in women than in men at baseline. Importantly, the sex effect on CSF Aβ42/40 was replicated in the WRAP/WADRC cohort. These findings suggest these biomarkers to be more indicative of Aβ pathology in the brain in women. As previous research has shown that women exhibit a higher pathological burden and faster disease progression during symptomatic stages [9, 10], it remains to be elucidated whether our findings reflect sex differences in the performance of biomarkers to accurately capture Aβ accumulation or, alternatively, they indicate a higher progression rate in CU women compared to men when initial pathophysiological changes are present. In addition, the stronger associations observed between glial biomarkers and Centiloid values in women compared to men (sTREM2, YKL-40 in ALFA+ and GFAP in WRAP/ADRC), further support a sex-specific glial response to AD pathology, in line with previous studies [38, 39]. Of note, although stratified analyses show stronger associations in women, the results of interactions between sex and CSF biomarkers on longitudinal change in Centiloid values did not reach significance after multiple comparison correction in ALFA+ and were not replicated in the WRAP/WADRC cohort. Further longitudinal studies with larger sample sizes and a longer follow-up period are warranted.

The results of this study have multiple implications on the accurate use of AD CSF biomarkers. First, they indicate that sex does not impact the use of core AD CSF biomarkers to identify Aβ PET-positive CU participants for preventive clinical trials. Second, if confirmed in further longitudinal studies, the stronger association of AD CSF biomarkers with Aβ burden measured by PET in women suggests that these biomarkers may be more effective in monitoring changes in Aβ deposition or treatment response to anti-Aβ therapies in women. Third, the knowledge of sex differences in the levels of CSF biomarkers for downstream processes contributes to our understanding of disease pathogenesis and can aid in the development of personalized treatment strategies, where the use of treatments targeting synaptic dysfunction might be more efficacious for women, while those targeting neurodegeneration and glial reactivity might be more efficacious for men.

## Strenghts and limitations

A key strength of our study is the inclusion of two similar and well-characterized cohorts with CSF biomarkers measured using the same analytical platforms, enhancing their comparability and the reliability of our findings. We examined core AD CSF biomarkers along with a wide range of CSF biomarkers reflecting Aβ-downstream processes, allowing for a comprehensive analysis of sex differences in different AD-related pathways. Different from other studies using sex as a covariate, herein we focused on the effect of sex and performed sex-stratified and interaction analyses. Nevertheless, our study has several limitations. The frequency of *APOE-*ε4 was higher in ALFA+ compared to WRAP/WADRC, and in ALFA+ the frequency of *APOE-*ε4 carriers was higher in men than in women. Yet, we adjusted all models by *APOE-*ε4 genotype and most results were replicated despite these differences. Also, some CSF biomarkers were only available in ALFA+, so we could not replicate the results on those. Moreover, the higher number of women than men enrolled in both cohorts may have limited the statistical power to detect significant effects in men. Finally, the study results could be influenced by factors that are beyond the scope of this study, such as menopause status, hormonal exposure, genetics, or differences in blood brain barrier permeability between men and women [28, 68]. Further research is warranted to evaluate their potential effects on biomarker levels and performance. Amid the recent development of blood biomarkers for AD, similar studies should be performed exploring their sex differences.

## Conclusions

Our study showed that, in two cohorts of CU individuals, there were no differences in the diagnostic accuracy of core AD CSF biomarkers. However, there were significant differences in the levels of CSF biomarkers reflecting Aβ-downstream processes, as well as how they were associated with disease pathophysiology as indicated by Aβ PET uptake. These findings highlight the importance of considering sex differences when using CSF biomarkers in preclinical AD.

## Supplementary Information

Additional file 1.

## Data Availability

The datasets used and/or analysed during the current study are available from the corresponding author on reasonable request.

## Abbreviations

Aβ: Amyloid-beta
AD: Alzheimer’s disease
ANCOVA: Analysis of covariance
ANOVA: Analysis of variance
APOE: Apolipoprotein E
AUC: Area under the curve
CSF: Cerebrospinal fluid
CU: Cognitively unimpaired
ERC: European Research Council
GAP-43: Growth-associated protein-43
GFAP: Glial fibrillary acidic protein
IL-6: Interleukin-6
MCP-1: Monocyte chemoattractant protein-1
MMSE: Mini-Mental State Examination
NfL: Neurofilament light
PACC: Preclinical Alzheimer’s Cognitive Composite
PET: Positron emission tomography
p-tau: Phosphorylated tau
ROC: Receiver-operating characteristic
sICAM-1: Soluble intercellular adhesion molecule-1
SNAP25: Synaptosomal-associated protein 25
sTREM2: Soluble triggering receptor expressed on myeloid cells 2
sVCAM-1: Soluble vascular cell adhesion molecule-1
t-tau: Total tau
YKL-40: Chitinase-3-like protein

## References

[1] Aisen PS, Cummings J, Jack CR, Morris JC, Sperling R, Frölich L, et al. On the path to 2025: understanding the Alzheimer’s disease continuum. Alzheimers Res Ther. 2017;9: 60. 28793924 10.1186/s13195-017-0283-5 PMC5549378

[2] Rafii MS, Aisen PS. Detection and treatment of Alzheimer’s disease in its preclinical stage. Nat Aging. 2023;3:520–31. 37202518 10.1038/s43587-023-00410-4 PMC11110912

[3] Jack CR. Advances in Alzheimer’s disease research over the past two decades. Lancet Neurol. 2022;21:866–9. 36115352 10.1016/S1474-4422(22)00298-8

[4] Milà-Alomà M, Salvadó G, Gispert JD, Vilor-Tejedor N, Grau-Rivera O, Sala-Vila A, et al. Amyloid beta, tau, synaptic, neurodegeneration, and glial biomarkers in the preclinical stage of the Alzheimer’s continuum. Alzheimers Dement. 2020;16:1358–71. 32573951 10.1002/alz.12131 PMC7586814

[5] Sutphen CL, Jasielec MS, Shah AR, Macy EM, Xiong C, Vlassenko AG, et al. Longitudinal cerebrospinal fluid biomarker changes in preclinical Alzheimer disease during middle age. JAMA Neurol. 2015;72:1029–42. 26147946 10.1001/jamaneurol.2015.1285 PMC4570860

[6] Rahman A, Jackson H, Hristov H, Isaacson RS, Saif N, Shetty T, et al. Sex and Gender Driven Modifiers of Alzheimer’s: The Role for Estrogenic Control Across Age, Race, Medical, and Lifestyle Risks. Frontiers in Aging Neuroscience. 2019;11:1–22. 31803046 10.3389/fnagi.2019.00315 PMC6872493

[7] Calvo N, Einstein G. Steroid hormones: risk and resilience in women’s Alzheimer disease. Front Aging Neurosci. 2023;15: 1159435. 37396653 10.3389/fnagi.2023.1159435 PMC10313425

[8] Eissman JM, Dumitrescu L, Mahoney ER, Smith AN, Mukherjee S, Lee ML, et al. Sex differences in the genetic architecture of cognitive resilience to Alzheimer’s disease. Brain. 2022;145:2541–54. 35552371 10.1093/brain/awac177 PMC9337804

[9] Barnes LL, Wilson RS, Bienias JL, Schneider JA, Evans DA, Bennett DA. Sex differences in the clinical manifestations of Alzheimer disease pathology. Arch Gen Psychiatry. 2005;62:685–91. 15939846 10.1001/archpsyc.62.6.685

[10] Holland D, Desikan RS, Dale AM, McEvoy LK. Higher rates of decline for women and Apolipoprotein E ε4 carriers. AJNR Am J Neuroradiol. 2013;34:2287–93. 23828104 10.3174/ajnr.A3601 PMC3894062

[11] Ferretti MT, Martinkova J, Biskup E, Benke T, Gialdini G, Nedelska Z, et al. Sex and gender differences in Alzheimer’s disease: current challenges and implications for clinical practice. Eur J Neurol. 2020;27:928–43. 32056347 10.1111/ene.14174

[12] Mielke MM, Ferretti MT, Iulita MF, Hayden K, Khachaturian AS. Sex and gender in Alzheimer’s disease - does it matter? Alzheimers Dement. 2018;14:1101–3. 30196887 10.1016/j.jalz.2018.08.003

[13] Bouter C, Vogelgsang J, Wiltfang J. Comparison between amyloid-PET and CSF amyloid-β biomarkers in a clinical cohort with memory deficits. Clin Chim Acta. 2019;492:62–8. 30735665 10.1016/j.cca.2019.02.005

[14] Buckley RF, Mormino EC, Chhatwal J, Schultz AP, Rabin JS, Rentz DM, et al. Associations between baseline amyloid, sex, and APOE on subsequent tau accumulation in cerebrospinal fluid. Neurobiol Aging. 2019;78:178–85. 30947113 10.1016/j.neurobiolaging.2019.02.019 PMC6545139

[15] Hohman TJ, Dumitrescu L, Barnes LL, Thambisetty M, Beecham G, Kunkle B, et al. Sex-specific association of apolipoprotein E with cerebrospinal fluid levels of tau. JAMA Neurol. 2018;75:989–98. 29801024 10.1001/jamaneurol.2018.0821 PMC6142927

[16] Li G, Shofer JB, Petrie EC, Yu CE, Wilkinson CW, Figlewicz DP, et al. Cerebrospinal fluid biomarkers for Alzheimer’s and vascular disease vary by age, gender, and APOE genotype in cognitively normal adults. Alzheimers Res Ther. 2017;9: 1–9. 28673336 10.1186/s13195-017-0271-9 PMC5496132

[17] Duarte-Guterman P, Albert AY, Barha CK, Galea LAM, On Behalf Of The Alzheimer’s Disease Neuroimaging Initiative null. Sex influences the effects of APOE genotype and Alzheimer’s diagnosis on neuropathology and memory. Psychoneuroendocrinology. 2021;129:105248. 10.1016/j.psyneuen.2021.105248 33962245

[18] Buckley RF, Scott MR, Jacobs HIL, Schultz AP, Properzi MJ, Amariglio RE, et al. Sex mediates relationships between regional tau pathology and cognitive decline. Ann Neurol. 2020;88:921–32. 32799367 10.1002/ana.25878 PMC7581543

[19] Koran MEI, Wagener M, Hohman TJ, Initiative for the AN. Sex differences in the association between AD biomarkers and cognitive decline. Brain Imaging and Behavior. 2017;11:205–13. 10.1007/s11682-016-9523-8 PMC4972701 26843008

[20] Altmann A, Tian L, Henderson VW, Greicius MD. Sex modifies the APOE-related risk of developing Alzheimer disease. Ann Neurol. 2014;75:563–73. 24623176 10.1002/ana.24135 PMC4117990

[21] Damoiseaux JS, Seeley WW, Zhou J, Shirer WR, Coppola G, Karydas A, et al. Gender modulates the APOE 4 effect in healthy older adults: convergent evidence from functional brain connectivity and spinal fluid tau levels. J Neurosci. 2012;32:8254–62. 22699906 10.1523/JNEUROSCI.0305-12.2012 PMC3394933

[22] Babapour Mofrad R, Tijms BM, Scheltens P, Barkhof F, van der Flier WM, Sikkes SAM, et al. Sex differences in CSF biomarkers vary by Alzheimer disease stage and APOE ε4 genotype. Neurology. 2020;95: e2378–88. 32788242 10.1212/WNL.0000000000010629

[23] Bridel C, van Wieringen WN, Zetterberg H, Tijms BM, Teunissen CE, Alvarez-Cermeño JC, et al. Diagnostic value of cerebrospinal fluid neurofilament light protein in neurology. JAMA Neurol. 2019;76:1035. 31206160 10.1001/jamaneurol.2019.1534 PMC6580449

[24] Delaby C, Alcolea D, Carmona-Iragui M, Illán-Gala I, Morenas-Rodríguez E, Barroeta I, et al. Differential levels of neurofilament light protein in cerebrospinal fluid in patients with a wide range of neurodegenerative disorders. Sci Rep. 2020;10: 9161. 32514050 10.1038/s41598-020-66090-x PMC7280194

[25] Lim B, Grøntvedt GR, Bathala P, Kale SS, Campbell CT, Stengelin M, et al. CSF neurofilament light may predict progression from amnestic mild cognitive impairment to Alzheimer’s disease dementia. Neurobiol Aging. 2021;107:78–85. 34403936 10.1016/j.neurobiolaging.2021.07.013

[26] Mattsson N, Insel PS, Palmqvist S, Portelius E, Zetterberg H, Weiner M, et al. Cerebrospinal fluid tau, neurogranin, and neurofilament light in Alzheimer’s disease. EMBO Mol Med. 2016;8:1184–96. 27534871 10.15252/emmm.201606540 PMC5048367

[27] Mielke MM, Syrjanen JA, Blennow K, Zetterberg H, Skoog I, Vemuri P, et al. Comparison of variables associated with cerebrospinal fluid neurofilament, total-tau, and neurogranin. Alzheimers Dement. 2019;15:1437–47. 31668594 10.1016/j.jalz.2019.07.009 PMC6874755

[28] Skillbäck T, Blennow K, Zetterberg H, Shams S, Machado A, Pereira J, et al. Sex differences in CSF biomarkers for neurodegeneration and blood-brain barrier integrity. Alzheimer’s & Dementia: Diagnosis, Assessment & Disease Monitoring. 2021;13: e12141. 10.1002/dad2.12141 PMC7968119 33748393

[29] Wang L, Alzheimer’s Disease Neuroimaging Initiative. Association of cerebrospinal fluid neurogranin with Alzheimer’s disease. Aging Clin Exp Res. 2019;31:185–91. 29667155 10.1007/s40520-018-0948-3

[30] Xue M, Sun F-R, Ou Y-N, Shen X-N, Li H-Q, Huang Y-Y, et al. Association of cerebrospinal fluid neurogranin levels with cognition and neurodegeneration in Alzheimer’s disease. Aging. 2020;12:9365–79. 32421689 10.18632/aging.103211 PMC7288926

[31] Agnello L, Gambino CM, Lo Sasso B, Bivona G, Milano S, Ciaccio AM, et al. Neurogranin as a novel biomarker in Alzheimer’s disease. Lab Med. 2021;52:188–96. 32926148 10.1093/labmed/lmaa062

[32] Casaletto KB, Elahi FM, Bettcher BM, Neuhaus J, Bendlin BB, Asthana S, et al. Neurogranin, a synaptic protein, is associated with memory independent of Alzheimer biomarkers. Neurology. 2017;89:1782–8. 28939668 10.1212/WNL.0000000000004569 PMC5664306

[33] Tarawneh R, D’Angelo G, Crimmins D, Herries E, Griest T, Fagan AM, et al. Diagnostic and prognostic utility of the synaptic marker neurogranin in Alzheimer disease. JAMA Neurol. 2016;73:561. 27018940 10.1001/jamaneurol.2016.0086 PMC4861689

[34] Duarte-Guterman P, Albert AY, Inkster AM, Barha CK, Galea LAM, Alzheimer’s Disease Neuroimaging Initiative. Inflammation in Alzheimer’s disease: do sex and APOE matter? J Alzheimers Dis. 2020;78:627–41. 33016923 10.3233/JAD-200982

[35] Janelidze S, Mattsson N, Stomrud E, Lindberg O, Palmqvist S, Zetterberg H, et al. CSF biomarkers of neuroinflammation and cerebrovascular dysfunction in early Alzheimer disease. Neurology. 2018;91:e867-77. 30054439 10.1212/WNL.0000000000006082 PMC6133624

[36] Brosseron F, Kolbe C, Santarelli F, Carvalho S, Antonell A, Castro-Gomez S, et al. Multicenter Alzheimer’s and Parkinson’s disease immune biomarker verification study. Alzheimers Dement. 2020;16:292–304. 31630996 10.1016/j.jalz.2019.07.018

[37] Nordengen K, Kirsebom B-E, Henjum K, Selnes P, Gísladóttir B, Wettergreen M, et al. Glial activation and inflammation along the Alzheimer’s disease continuum. J Neuroinflammation. 2019;16: 46. 30791945 10.1186/s12974-019-1399-2 PMC6383268

[38] Salvadó G, Milà-Alomà M, Shekari M, Minguillon C, Fauria K, Niñerola-Baizán A, et al. Cerebral amyloid-β load is associated with neurodegeneration and gliosis: mediation by p-tau and interactions with risk factors early in the Alzheimer’s continuum. Alzheimers Dement. 2021;17:788–800. 33663013 10.1002/alz.12245 PMC8252618

[39] Vila-Castelar C, Akinci M, Palpatzis E, Aguilar-Dominguez P, Operto G, Kollmorgen G, et al. Sex/gender effects of glial reactivity on preclinical Alzheimer’s disease pathology. Mol Psychiatry. 2025;30:1430–9. 39384963 10.1038/s41380-024-02753-9 PMC11919761

[40] Molinuevo JL, Gramunt N, Gispert JD, Fauria K, Esteller M, Minguillon C, et al. The ALFA project: a research platform to identify early pathophysiological features of Alzheimer’s disease. Alzheimers Dement (N Y). 2016;2:82–92. 29067295 10.1016/j.trci.2016.02.003 PMC5644283

[41] Johnson SC, Koscik RL, Jonaitis EM, Clark LR, Mueller KD, Berman SE, et al. The Wisconsin registry for Alzheimer’s prevention: a review of findings and current directions. Alzheimer’s & Dementia: Diagnosis, Assessment & Disease Monitoring. 2018;10:130–42. 10.1016/j.dadm.2017.11.007 PMC5755749 29322089

[42] Bettcher BM, Johnson SC, Fitch R, Casaletto KB, Heffernan KS, Asthana S, et al. CSF and Plasma Levels of Inflammation Differentially Relate to CNS Markers of Alzheimer’s Disease Pathology and Neuronal Damage HHS Public Access. J Alzheimers Dis. 2018;62:385–97. 29439331 10.3233/JAD-170602 PMC6007886

[43] Folstein MF, Folstein SE, McHugh PR. “Mini-mental state”. A practical method for grading the cognitive state of patients for the clinician. J Psychiatr Res. 1975;12:189–98. 1202204 10.1016/0022-3956(75)90026-6

[44] Papp KV, Rentz DM, Orlovsky I, Sperling RA, Mormino EC. Optimizing the preclinical Alzheimer’s cognitive composite with semantic processing: The PACC5. Alzheimers Dement (N Y). 2017;3:668–77. 29264389 10.1016/j.trci.2017.10.004 PMC5726754

[45] Donohue MC, Sperling RA, Petersen R, Sun C-K, Weiner MW, Aisen PS. Association between elevated brain amyloid and subsequent cognitive decline among cognitively normal persons. JAMA. 2017;317:2305. 28609533 10.1001/jama.2017.6669 PMC5736301

[46] Suárez-Calvet M, Karikari TK, Ashton NJ, Lantero Rodríguez J, Milà-Alomà M, Gispert JD, et al. Novel tau biomarkers phosphorylated at T181, T217 or T231 rise in the initial stages of the preclinical Alzheimer’s continuum when only subtle changes in Aβ pathology are detected. EMBO Mol Med. 2020;12:1–19. 10.15252/emmm.202012921 PMC7721364 33169916

[47] Van Hulle C, Jonaitis EM, Betthauser TJ, Batrla R, Wild N, Kollmorgen G, et al. An examination of a novel multipanel of CSF biomarkers in the Alzheimer’s disease clinical and pathological continuum. Alzheimers Dement. 2021;17:431-445. 33336877 10.1002/alz.12204 PMC8016695

[48] Ashton NJ, Pascoal TA, Karikari TK, Benedet AL, Lantero-Rodriguez J, Brinkmalm G, et al. Plasma p-tau231: a new biomarker for incipient Alzheimer’s disease pathology. Acta Neuropathol. 2021;141:709–24. 33585983 10.1007/s00401-021-02275-6 PMC8043944

[49] Thijssen EH, La Joie R, Strom A, Fonseca C, Iaccarino L, Wolf A, et al. Plasma phosphorylated tau 217 and phosphorylated tau 181 as biomarkers in Alzheimer’s disease and frontotemporal lobar degeneration: a retrospective diagnostic performance study. Lancet Neurol. 2021;20:739–52. 34418401 10.1016/S1474-4422(21)00214-3 PMC8711249

[50] Tible M, Sandelius Å, Höglund K, Brinkmalm A, Cognat E, Dumurgier J, et al. Dissection of synaptic pathways through the CSF biomarkers for predicting Alzheimer disease. Neurology. 2020;95:e953-61. 32586895 10.1212/WNL.0000000000010131

[51] Sandelius Å, Portelius E, Källén Å, Zetterberg H, Rot U, Olsson B, et al. Elevated CSF GAP-43 is Alzheimer’s disease specific and associated with tau and amyloid pathology. Alzheimers Dement. 2019;15:55–64. 30321501 10.1016/j.jalz.2018.08.006 PMC6333489

[52] Shekari M, Vállez García D, Collij LE, Altomare D, Heeman F, Pemberton H, et al. Stress testing the Centiloid: Precision and variability of PET quantification of amyloid pathology. Alzheimers Dement. 2024;20:5102–13. 38961808 10.1002/alz.13883 PMC11350134

[53] Johnson SC, Christian BT, Okonkwo OC, Oh JM, Harding S, Xu G, et al. Amyloid burden and neural function in people at risk for Alzheimer’s disease. Neurobiol Aging. 2014;35:576–84. 24269021 10.1016/j.neurobiolaging.2013.09.028 PMC4018215

[54] Betthauser TJ, Bilgel M, Koscik RL, Jedynak BM, An Y, Kellett KA, et al. Multi-method investigation of factors influencing amyloid onset and impairment in three cohorts. Brain. 2022;145:4065–79. 35856240 10.1093/brain/awac213 PMC9679170

[55] Klunk WE, Koeppe RA, Price JC, Benzinger TL, Devous MD, Jagust WJ, et al. The centiloid project: standardizing quantitative amyloid plaque estimation by PET. Alzheimers Dement. 2015;11:1-15.e4. 25443857 10.1016/j.jalz.2014.07.003 PMC4300247

[56] Rea Reyes RE, Cody KA, Wilson RE, Zetterberg H, Chin NA, Jonaitis EM, et al. Visual read of [F-18]florquinitau PET that includes and extends beyond the mesial temporal lobe is associated with increased plasma pTau217 and cognitive decline in a cohort that is enriched with risk for Alzheimer’s disease. Alzheimers Dement. 2025;21:e14406. 39560002 10.1002/alz.14406 PMC11848396

[57] Benjamini Y, Hochberg Y. Controlling the false discovery rate: a practical and powerful approach to multiple testing. J R Stat Soc Series B Stat Methodol. 1995;57:289–300.

[58] Janelidze S, Zetterberg H, Mattsson N, Palmqvist S, Vanderstichele H, Lindberg O, et al. CSF A β 42/A β 40 and A β 42/A β 38 ratios: better diagnostic markers of Alzheimer disease. Ann Clin Transl Neurol. 2016;3:154–65. 27042676 10.1002/acn3.274 PMC4774260

[59] Jack CR, Andrews JS, Beach TG, Buracchio T, Dunn B, Graf A, et al. Revised criteria for diagnosis and staging of Alzheimer’s disease: Alzheimer’s association workgroup. Alzheimers Dement. 2024;20:5143–5169. 38934362 10.1002/alz.13859 PMC11350039

[60] Therriault J, Vermeiren M, Servaes S, Tissot C, Ashton NJ, Benedet AL, et al. Association of phosphorylated tau biomarkers with amyloid positron emission tomography vs tau positron emission tomography. JAMA Neurol. 2023;80:188–199. 10.1001/jamaneurol.2022.4485 PMC9856704 36508198

[61] Mattsson-Carlgren N, Janelidze S, Bateman RJ, Smith R, Stomrud E, Serrano GE, et al. Soluble P-tau217 reflects amyloid and tau pathology and mediates the association of amyloid with tau. EMBO Mol Med. 2021;13: e14022. 33949133 10.15252/emmm.202114022 PMC8185545

[62] Mielke MM, Hagen CE, Xu J, Chai X, Vemuri P, Lowe VJ, et al. Plasma phospho-tau181 increases with Alzheimer’s disease clinical severity and is associated with tau- and amyloid-positron emission tomography. Alzheimers Dement. 2018;14:989–97. 29626426 10.1016/j.jalz.2018.02.013 PMC6097897

[63] Murray ME, Moloney CM, Kouri N, Syrjanen JA, Matchett BJ, Rothberg DM, et al. Global neuropathologic severity of Alzheimer’s disease and locus coeruleus vulnerability influences plasma phosphorylated tau levels. Mol Neurodegener. 2022;17: 85. 36575455 10.1186/s13024-022-00578-0 PMC9795667

[64] Coughlan GT, Betthauser TJ, Boyle R, Koscik RL, Klinger HM, Chibnik LB, et al. Association of age at menopause and hormone therapy use with tau and β-amyloid positron emission tomography. JAMA Neurol. 2023;80:462–73. 37010830 10.1001/jamaneurol.2023.0455 PMC10071399

[65] Sundermann EE, Panizzon MS, Chen X, Andrews M, Galasko D, Banks SJ. Sex differences in Alzheimer’s-related tau biomarkers and a mediating effect of testosterone. Biol Sex Differ. 2020;11: 33. 32560743 10.1186/s13293-020-00310-x PMC7304096

[66] Gur RC, Turetsky BI, Matsui M, Yan M, Bilker W, Hughett P, et al. Sex differences in brain gray and white matter in healthy young adults: correlations with cognitive performance. J Neurosci. 1999;19:4065–72. 10234034 10.1523/JNEUROSCI.19-10-04065.1999 PMC6782697

[67] Milà-Alomà M, Brinkmalm A, Ashton NJ, Kvartsberg H, Shekari M, Operto G, et al. CSF synaptic biomarkers in the preclinical stage of Alzheimer disease and their association with MRI and PET. Neurology. 2021;97:e2065–78. 34556565 10.1212/WNL.0000000000012853 PMC8610620

[68] Parrado-Fernández C, Blennow K, Hansson M, Leoni V, Cedazo-Minguez A, Björkhem I. Evidence for sex difference in 30054982 10.1111/jcmm.13767 PMC6156389

