# Supplementary material for: Sex differences in Alzheimer’s disease CSF biomarkers and their association with Aβ pathology on PET in cognitively unimpaired individuals
Source: Alzheimers Res Ther. 2025 Oct 30;17:235. doi: 10.1186/s13195-025-01844-1 (PMC12573942; doi:10.1186/s13195-025-01844-1)
Supplement: Supplementary file 1 — Additional file 1. [file 13195_2025_1844_MOESM1_ESM.docx]

**Supplementary material**

**Table of Contents**

[Supplementary Table 1. Modifying effect of CSF Aβ status on the association between sex and baseline CSF biomarkers 2](#_Toc207181226)

[Supplementary Table 2. Modifying effect of sex in the association between baseline CSF biomarkers and baseline Centiloid values 3](#_Toc207181227)

[Supplementary Table 3. Effect of baseline CSF biomarkers on baseline Centiloid values by sex 4](#_Toc207181228)

[Supplementary Table 4. Modifying effect of sex in the association between baseline CSF biomarkers and change in Centiloid values at follow-up 5](#_Toc207181229)

[Supplementary Table 5. Effect of baseline CSF biomarkers on change in Centiloid values at follow-up by sex 6](#_Toc207181230)

[Supplementary Table 6. Modifying effect of sex in the association between baseline CSF biomarkers and change in Centiloid values at follow-up (subset with baseline Centiloid < 30) 7](#_Toc207181231)

[Supplementary Figure 1. Associations between baseline core AD CSF biomarkers and change in Centiloid values at follow-up by sex 8](#_Toc207181232)

[Supplementary Figure 2. Associations between baseline non-AD-specific CSF biomarkers and change in Centiloid values at follow-up by sex 9](#_Toc207181233)

# Supplementary Table 1. Modifying effect of CSF Aβ status on the association between sex and baseline CSF biomarkers

|  | **Interaction: CSF Aβ status** $\boldsymbol{\times}$ **sex** | | | |
| --- | --- | --- | --- | --- |
|  | **ALFA+** | | **WRAP/ADRC** | |
| **Outcome: Baseline CSF biomarker** | **β (SE)** | ***P*-value** | **β (SE)** | ***P*-value** |
| **A**β**42** | -0.04 (0.21) | 0.84 | -0.14 (0.22) | 0.52 |
| **A**β**42/40** | -0.05 (0.11) | 0.67 | -0.13 (0.15) | 0.48 |
| **p-tau181/A**β**42** | 0.14 (0.20) | 0.49 | 0.11 (0.22) | 0.60 |
| **p-tau181** | 0.23 (0.20) | 0.25 | 0.38 (0.15) | **0.020** |
| **p-tau217** | 0.28 (0.18) | 0.13 |  |  |
| **p-tau231** | 0.30 (0.18) | 0.10 |  |  |
| **t-tau** | 0.18 (0.20) | 0.39 | 0.16 (0.22) | 0.63 |
| **NfL** | 0.21 (0.19) | 0.27 | 0.19 (0.19) | 0.42 |
| **neurogranin** | 0.16 (0.21) | 0.45 | -0.05 (0.24) | 0.82 |
| **GAP-43** | 0.06 (0.21) | 0.77 |  |  |
| **SNAP25** | 0.21 (0.21) | 0.33 |  |  |
| **synaptotagmin-1** | 0.25 (0.21) | 0.24 |  |  |
| **α−synuclein** | 0.25 (0.22) | 0.25 | 0.18 (0.24) | 0.46 |
| **GFAP** | 0.36 (0.20) | 0.07 | 0.29 (0.24) | 0.32 |
| **S100B** | 0.19 (0.21) | 0.35 | -0.16 (0.21) | 0.46 |
| **sTREM2** | 0.38 (0.21) | 0.08 | 0.30 (0.24) | 0.18 |
| **YKL-40** | 0.36 (0.20) | 0.07 | -0.26 (0.24) | 0.29 |
| **IL-6** | 0.02 (0.22) | 0.94 | 0.43 (0.25) | 0.09 |
| **MCP-1** | -0.18 (0.22) | 0.42 |  |  |
| **sICAM-1** | 0.19 (0.22) | 0.37 |  |  |
| **sVCAM-1** | 0.11 (0.21) | 0.60 |  |  |

Interaction terms between sex and CSF Aβ42/40 status were added in independent regression models adjusted by age and *APOE*-ε4 status with each baseline CSF biomarker as the outcome. Beta coefficients (β), standard errors (SE) and *P*-values for the interaction term are reported. *P*-values were not significant after correction for multiple comparisons using FDR.

Abbreviations: Aβ40, amyloid-β 40; Aβ42, amyloid-β 42; GAP-43; growth-associated protein-43; GFAP, glial fibrillary acidic protein; IL-6, interleukin 6; MCP-1, monocyte chemoattractant protein-1; NfL, neurofilament light; p-tau, phosphorylated tau; sICAM-1, soluble intercellular adhesion molecule-1; SNAP25; synaptosomal-associated protein 25; sTREM2, soluble triggering receptor expressed on myeloid cells 2; sVCAM-1, soluble vascular cell adhesion molecule-1; t-tau, total tau; YKL-40, chitinase-3-like protein 1.

# Supplementary Table 2. Modifying effect of sex in the association between baseline CSF biomarkers and baseline Centiloid values

|  | **Interaction: CSF biomarker** $\boldsymbol{\times}$ **sex** | | | |
| --- | --- | --- | --- | --- |
|  | **ALFA+** | | **WRAP/ADRC** | |
| **Outcome: Baseline Centiloid** | **β (SE)** | ***P*-value** | **β (SE)** | ***P*-value** |
| **A**β**42** | 0.05 (0.07) | 0.45 | 0.18 (0.14) | 0.23 |
| **A**β**42/40** | 0.11 (0.04) | **0.012*** | 0.35 (0.12) | **0.003*** |
| **p-tau181/A**β**42** | -0.29 (0.10) | **0.005*** | -0.02 (0.13) | 0.85 |
| **p-tau181** | -0.12 (0.05) | **0.022** | -0.28 (0.15) | 0.078 |
| **p-tau217** | -0.11 (0.04) | **0.011*** |  |  |
| **p-tau231** | -0.14 (0.04) | **0.001*** |  |  |
| **t-tau** | -0.12 (0.05) | **0.021** | -0.26 (0.16) | 0.11 |
| **NfL** | -0.18 (0.06) | **0.001*** | -0.31 (0.16) | 0.068 |
| **neurogranin** | -0.10 (0.05) | 0.060 | -0.21 (016) | 0.17 |
| **GAP-43** | -0.08 (0.05) | 0.15 |  |  |
| **SNAP25** | -0.11 (0.06) | 0.055 |  |  |
| **synaptotagmin-1** | -0.11 (0.06) | 0.053 |  |  |
| **α−synuclein** | -0.09 (0.06) | 0.12 | -0.32 (0.16) | **0.048** |
| **GFAP** | -0.07 (0.05) | 0.18 | -0.36 (0.16) | **0.025*** |
| **S100B** | -0.03 (0.05) | 0.60 | -0.14 (0.16) | 0.38 |
| **sTREM2** | -0.14 (0.05) | **0.013*** | -0.12 (0.17) | 0.47 |
| **YKL-40** | -0.15 (0.05) | **0.005*** | -0.08 (0.16) | 0.63 |
| **IL-6** | 0.01 (0.06) | 0.88 | -0.16 (0.16) | 0.37 |
| **MCP-1** | -0.04 (0.06) | 0.53 |  |  |
| **sICAM-1** | -0.14 (0.06) | **0.022** |  |  |
| **sVCAM-1** | -0.13 (0.06) | **0.028** |  |  |

Interaction terms between each baseline CSF biomarker and sex were added in independent regression models adjusted by age and *APOE*-ε4 status with baseline Centiloid values as the outcome. Beta coefficients (β), standard errors (SE) and *P*-values for the interaction term are reported. Significant *P*-values are shown in bold. *Significant *P*-values after correction for multiple comparisons using FDR.

Abbreviations: Aβ40, amyloid-β 40; Aβ42, amyloid-β 42; GAP-43; growth-associated protein-43; GFAP, glial fibrillary acidic protein; IL-6, interleukin 6; MCP-1, monocyte chemoattractant protein-1; NfL, neurofilament light; p-tau, phosphorylated tau; sICAM-1, soluble intercellular adhesion molecule-1; SNAP25; synaptosomal-associated protein 25; sTREM2, soluble triggering receptor expressed on myeloid cells 2; sVCAM-1, soluble vascular cell adhesion molecule-1; t-tau, total tau; YKL-40, chitinase-3-like protein 1.

# Supplementary Table 3. Effect of baseline CSF biomarkers on baseline Centiloid values by sex

|  | **ALFA+** | | | | **WRAP/ADRC** | | | |
| --- | --- | --- | --- | --- | --- | --- | --- | --- |
|  | **Women** | | **Men** | | **Women** | | **Men** | |
| **Outcome:**  **Baseline Centiloid** | **β (SE)** | ***P*-value** | **β (SE)** | ***P*-value** | **β (SE)** | ***P*-value** | **β (SE)** | ***P*-value** |
| **A**β**42** | -0.41 (0.10) | **<0.001*** | -0.40 (0.11) | **<0.001*** | -0.47 (0.08) | **<0.001*** | -0.42 (0.13) | **0.001*** |
| **A**β**42/40** | -0.66 (0.06) | **<0.001*** | -0.53 (0.08) | **<0.001*** | -0.71 (0.07) | **<0.001*** | -0.61 (0.12) | **<0.001*** |
| **p-tau181/A**β**42** | 0.78 (0.07) | **<0.001*** | 0.60 (0.09) | **<0.001*** | 0.76 (0.07) | **<0.001*** | 0.74 (0.09) | **<0.001*** |
| **p-tau181** | 0.35 (0.06) | **<0.001*** | 0.23 (0.08) | **0.006*** | 0.42 (0.08) | **<0.001*** | 0.28 (0.14) | 0.05 |
| **p-tau217** | 0.53 (0.05) | **<0.001*** | 0.48 (0.07) | **<0.001*** |  |  |  |  |
| **p-tau231** | 0.63 (0.05) | **<0.001*** | 0.52 (0.08) | **<0.001*** |  |  |  |  |
| **t-tau** | 0.32 (0.07) | **<0.001*** | 0.24 (0.09) | **0.006*** | 0.33 (0.09) | **<0.001*** | 0.18 (0.14) | 0.20 |
| **NfL** | 0.31 (0.07) | **<0.001*** | 0.12 (0.09) | 0.21 | 0.11 (0.10) | 0.29 | -0.13 (0.18) | 0.47 |
| **neurogranin** | 0.20 (0.07) | **0.003*** | 0.12 (0.09) | 0.16 | 0.20 (0.08) | **0.02*** | 0.04 (0.13) | 0.75 |
| **GAP-43** | 0.19 (0.06) | **0.003*** | 0.14 (0.09) | 0.11 |  |  |  |  |
| **SNAP25** | 0.18 (0.07) | **0.007*** | 0.10 (0.09) | 0.28 |  |  |  |  |
| **synaptotagmin-1** | 0.19 (0.07) | **0.006*** | 0.09 (0.09) | 0.33 |  |  |  |  |
| **α−synuclein** | 0.11 (0.07) | 0.11 | 0.05 (0.10) | 0.58 | 0.17 (0.09) | 0.05 | -0.14 (0.13) | 0.29 |
| **GFAP** | 0.21 (0.07) | **0.003*** | 0.12 (0.09) | 0.17 | 0.13 (0.11) | 0.22 | -0.22 (0.15) | 0.16 |
| **S100B** | 0.11 (0.07) | 0.092 | 0.00 (0.09) | 0.99 | 0.05 (0.09) | 0.65 | -0.10 (0.0.9) | 0.46 |
| **sTREM2** | 0.19 (0.07) | **0.008*** | -0.07 (0.09) | 0.51 | -0.11 (0.09) | 0.22 | -0.22 (0.15) | 0.14 |
| **YKL-40** | 0.29 (0.07) | **<0.001*** | 0.05 (0.10) | 0.63 | -0.10 (0.13) | 0.46 | -0.12 (0.15) | 0.41 |
| **IL-6** | 0.03 (0.07) | 0.66 | 0.10 (0.09) | 0.28 | 0.06 (0.09) | 0.53 | -0.13 (0.13) | 0.33 |
| **MCP-1** | 0.01 (0.07) | 0.85 | -0.04 (0.09) | 0.66 |  |  |  |  |
| **sICAM-1** | 0.18 (0.08) | **0.02*** | -0.00 (0.10) | 0.97 |  |  |  |  |
| **sVCAM-1** | 0.13 (0.07) | 0.07 | -0.03 (0.09) | 0.73 |  |  |  |  |

Main effects of each baseline CSF biomarker on baseline Centiloid values were evaluated in independent regression models stratified by sex and adjusted by age and *APOE*-ε4 status. Beta coefficients (β), standard errors (SE) and *P*-values are reported. Significant *P*-values are shown in bold. *Significant *P*-values after correction for multiple comparisons using FDR.

Abbreviations: Aβ40, amyloid-β 40; Aβ42, amyloid-β 42; GAP-43; growth-associated protein-43; GFAP, glial fibrillary acidic protein; IL-6, interleukin 6; MCP-1, monocyte chemoattractant protein-1; NfL, neurofilament light; p-tau, phosphorylated tau; sICAM-1, soluble intercellular adhesion molecule-1; SNAP25; synaptosomal-associated protein 25; sTREM2, soluble triggering receptor expressed on myeloid cells 2; sVCAM-1, soluble vascular cell adhesion molecule-1; t-tau, total tau; YKL-40, chitinase-3-like protein 1.

# Supplementary Table 4. Modifying effect of sex in the association between baseline CSF biomarkers and change in Centiloid values at follow-up

|  | **Interaction: CSF biomarker** $\boldsymbol{\times}$ **sex** | | | |
| --- | --- | --- | --- | --- |
|  | **ALFA+** | | **WRAP/ADRC** | |
| **Outcome: Change in Centiloid** | **β (SE)** | ***P*-value** | **β (SE)** | ***P*-value** |
| **A**β**42** | 0.10 (0.08) | 0.21 | -0.19 (0.14) | 0.17 |
| **A**β**42/40** | 0.15 (0.06) | **0.008** | -0.02 (0.12) | 0.86 |
| **p-tau181/A**β**42** | -0.26 (0.09) | **0.006** | 0.19 (0.12) | 0.12 |
| **p-tau181** | -0.08 (0.07) | 0.26 | -0.14 (0.14) | 0.35 |
| **p-tau217** | -0.13 (0.06) | **0.033** |  |  |
| **p-tau231** | -0.13 (0.06) | **0.035** |  |  |
| **t-tau** | -0.08 (0.07) | 0.22 | -0.12 (0.15) | 0.41 |
| **NfL** | -0.16 (0.08) | **0.031** | -0.15 (0.15) | 0.33 |
| **neurogranin** | -0.06 (0.07) | 0.43 | -0.16 0.15) | 0.29 |
| **GAP-43** | -0.07 (0.07) | 0.31 |  |  |
| **SNAP25** | 0.01 (0.07) | 0.85 |  |  |
| **synaptotagmin-1** | -0.06 (0.07) | 0.40 |  |  |
| **α−synuclein** | -0.01 (0.07) | 0.85 | -0.26 (0.15) | 0.09 |
| **GFAP** | -0.08 (0.07) | 0.26 | -0.06 (0.15) | 0.68 |
| **S100B** | -0.027 (0.067) | 0.69 | -0.12 (0.15) | 0.50 |
| **sTREM2** | -0.03 (0.07) | 0.65 | -0.21 (0.16) | 0.18 |
| **YKL-40** | -0.13 (0.07) | 0.06 | -0.03 (0.14) | 0.81 |
| **IL-6** | 0.05 (0.07) | 0.50 | 0.03 (0.15) | 0.87 |
| **MCP-1** | 0.02 (0.07) | 0.75 |  |  |
| **sICAM-1** | -0.07 (0.08) | 0.36 |  |  |
| **sVCAM-1** | -0.06 (0.08) | 0.45 |  |  |

Interaction terms between each baseline CSF biomarker and sex were added in independent regression models adjusted by age, *APOE*-ε4 status and time difference between baseline and follow-up visits, with change in Centiloids at follow-up as the outcome. Beta coefficients (β), standard errors (SE) and *P*-values for the interaction term are reported. *P*-values were not significant after correction for multiple comparisons using FDR.

Abbreviations: Aβ40, amyloid-β 40; Aβ42, amyloid-β 42; GAP-43; growth-associated protein-43; GFAP, glial fibrillary acidic protein; IL-6, interleukin 6; MCP-1, monocyte chemoattractant protein-1; NfL, neurofilament light; p-tau, phosphorylated tau; sICAM-1, soluble intercellular adhesion molecule-1; SNAP25; synaptosomal-associated protein 25; sTREM2, soluble triggering receptor expressed on myeloid cells 2; sVCAM-1, soluble vascular cell adhesion molecule-1; t-tau, total tau; YKL-40, chitinase-3-like protein 1.

# Supplementary Table 5. Effect of baseline CSF biomarkers on change in Centiloid values at follow-up by sex

|  | **ALFA+** | | | | **WRAP/ADRC** | | | |
| --- | --- | --- | --- | --- | --- | --- | --- | --- |
|  | **Women** | | **Men** | | **Women** | | **Men** | |
| **Outcome:**  **Change in Centiloid** | **β (SE)** | ***P*-value** | **β (SE)** | ***P*-value** | **β (SE)** | ***P*-value** | **β (SE)** | ***P*-value** |
| **A**β**42** | -0.34 (0.11) | **0.002*** | -0.17 (0.15) | 0.27 | **-0.21 (0.08)** | **0.008*** | -0.38 (0.14) | **0.012*** |
| **A**β**42/40** | -0.60 (0.07) | **<0.001*** | -0.39 (0.12) | **0.002*** | -0.45 (0.07) | **<0.001*** | -0.46 (0.13) | **0.001*** |
| **p-tau181/A**β**42** | 0.61 (0.09) | **<0.001*** | 0.33 (0.15) | **0.031** | 0.43 (0.07) | **<0.001*** | 0.62 (0.11) | **<0.001*** |
| **p-tau181** | 0.25 (0.08) | **<0.003*** | 0.12 (0.12) | 0.32 | 0.27 (0.08) | **0.001*** | 0.16 (0.15) | 0.28 |
| **p-tau217** | 0.47 (0.07) | **<0.001*** | 0.30 (0.12) | **0.019** |  |  |  |  |
| **p-tau231** | 0.47 (0.07) | **<0.001*** | 0.33 (0.12) | **0.007*** |  |  |  |  |
| **t-tau** | 0.23 (0.08) | **0.005*** | 0.13 (0.12) | 0.32 | 0.24 (0.08) | **0.004*** | 0.15 (0.15) | 0.32 |
| **NfL** | 0.33 (0.09) | **<0.001*** | 0.13 (0.14) | 0.36 | 0.05 (0.10) | 0.59 | -0.12 (0.17) | 0.50 |
| **neurogranin** | 0.13 (0.08) | 0.12 | 0.05 (0.12) | 0.69 | 0.18 (0.08) | **0.02*** | 0.07 (0.14) | 0.61 |
| **GAP-43** | 0.13 (0.08) | 0.11 | 0.03 (0.13) | 0.84 |  |  |  |  |
| **SNAP25** | 0.10 (0.08) | 0.21 | 0.24 (0.13) | 0.08 |  |  |  |  |
| **synaptotagmin-1** | 0.10 (0.08) | 0.19 | 0.02 (0.11) | 0.90 |  |  |  |  |
| **α−synuclein** | 0.04 (0.08) | 0.65 | 0.01 (0.12) | 0.95 | 0.10 (0.08) | 0.24 | -0.14 (0.14) | 0.31 |
| **GFAP** | 0.17 (0.09) | 0.050 | 0.09 (0.12) | 0.44 | 0.06 (0.10) | 0.55 | 0.06 (0.16) | 0.72 |
| **S100B** | 0.09 (0.08) | 0.28 | 0.07 (0.12) | 0.58 | 0.00 (0.08) | 0.96 | -0.19 (0.15) | 0.22 |
| **sTREM2** | -0.02 (0.09) | 0.84 | -0.10 (0.13) | 0.44 | 0.11 (0.08) | 0.18 | -0.08 (0.17) | 0.65 |
| **YKL-40** | 0.24 (0.10) | **0.011*** | 0.01 (0.14) | 0.96 | -0.05 (0.09) | 0.59 | -0.10 (0.16) | 0.52 |
| **IL-6** | 0.06 (0.08) | 0.50 | 0.13 (0.13) | 0.32 | -0.04 (0.08) | 0.61 | -0.03 (0.15) | 0.88 |
| **MCP-1** | -0.08 (0.09) | 0.40 | -0.01 (0.12) | 0.92 |  |  |  |  |
| **sICAM-1** | 0.06 (0.10) | 0.58 | 0.00 (0.15) | 0.99 |  |  |  |  |
| **sVCAM-1** | 0.06 (0.10) | 0.54 | 0.04 (0.13) | 0.80 |  |  |  |  |

Main effects of each baseline CSF biomarker on change in Centiloid values at follow-up were evaluated in independent regression models stratified by sex and adjusted by age, *APOE*-ε4 status and time difference between baseline and follow-up visits. Beta coefficients (β), standard errors (SE) and *P*-values are reported. Significant *P*-values are shown in bold. *Significant *P*-values after correction for multiple comparisons using FDR.

Abbreviations: Aβ40, amyloid-β 40; Aβ42, amyloid-β 42; GAP-43; growth-associated protein-43; GFAP, glial fibrillary acidic protein; IL-6, interleukin 6; MCP-1, monocyte chemoattractant protein-1; NfL, neurofilament light; p-tau, phosphorylated tau; sICAM-1, soluble intercellular adhesion molecule-1; SNAP25; synaptosomal-associated protein 25; sTREM2, soluble triggering receptor expressed on myeloid cells 2; sVCAM-1, soluble vascular cell adhesion molecule-1; t-tau, total tau; YKL-40, chitinase-3-like protein 1.

# Supplementary Table 6. Modifying effect of sex in the association between baseline CSF biomarkers and change in Centiloid values at follow-up (subset with baseline Centiloid < 30)

|  | **Interaction: CSF biomarker** $\boldsymbol{\times}$ **sex** | | | |
| --- | --- | --- | --- | --- |
|  | **ALFA+**  (n=190; 112 [58.9 %] women) | | **WRAP/ADRC**  (n=123; 82 [66.7 %] women) | |
| **Outcome: Change in Centiloid** | **β (SE)** | ***P*-value** | **β (SE)** | ***P*-value** |
| **A**β**42** | 0.12 (0.09) | 0.18 | -0.24 (0.14) | 0.12 |
| **A**β**42/40** | 0.22 (0.07) | **0.003** | 0.02 (0.18) | 0.88 |
| **p-tau181/A**β**42** | -0.23 (0.09) | **0.013** | 0.10 (0.18) | 0.28 |
| **p-tau181** | -0.07 (0.07) | 0.32 | -0.34 (0.19) | 0.26 |
| **p-tau217** | -0.12 (0.07) | 0.070 |  |  |
| **p-tau231** | -0.14 (0.07) | **0.040** |  |  |
| **t-tau** | -0.08 (0.07) | 0.28 | -0.26 (0.20) | 0.42 |
| **NfL** | -0.11 (0.08) | 0.18 | -0.28 (0.19) | 0.61 |
| **neurogranin** | -0.05 (0.08) | 0.49 | -0.37 0.20) | 0.34 |
| **GAP-43** | -0.08 (0.08) | 0.30 |  |  |
| **SNAP25** | 0.26 (0.08) | 0.74 |  |  |
| **synaptotagmin-1** | -0.05 (0.07) | 0.47 |  |  |
| **α−synuclein** | -0.019 (0.07) | 0.79 | -0.43 (0.18) | 0.32 |
| **GFAP** | -0.05 (0.08) | 0.55 | -0.22 (0.20) | 0.86 |
| **S100B** | -0.03 (0.07) | 0.72 | -0.15 (0.18) | 0.39 |
| **sTREM2** | 0.32 (0.07) | 0.67 | -0.22 (0.18) | 0.23 |
| **YKL-40** | -0.10 (0.08) | 0.18 | -0.13 (0.17) | 0.42 |
| **IL-6** | -0.00 (0.07) | 0.98 | 0.09 (0.16) | 0.59 |
| **MCP-1** | 0.07 (0.08) | 0.42 |  |  |
| **sICAM-1** | -0.02 (0.09) | 0.79 |  |  |
| **sVCAM-1** | -0.00 (0.09) | 0.99 |  |  |

Interaction terms between each baseline CSF biomarker and sex were added in independent regression models adjusted by age, *APOE*-ε4 status and time difference between baseline and follow-up visits, with change in Centiloids at follow-up as the outcome. Baseline Centiloid values were restricted to lower than 30 for these analyses. Beta coefficients (β), standard errors (SE) and *P*-values for the interaction term are reported. *P*-values were not significant after correction for multiple comparisons using FDR.

Abbreviations: Aβ40, amyloid-β 40; Aβ42, amyloid-β 42; GAP-43; growth-associated protein-43; GFAP, glial fibrillary acidic protein; IL-6, interleukin 6; MCP-1, monocyte chemoattractant protein-1; NfL, neurofilament light; p-tau, phosphorylated tau; sICAM-1, soluble intercellular adhesion molecule-1; SNAP25; synaptosomal-associated protein 25; sTREM2, soluble triggering receptor expressed on myeloid cells 2; sVCAM-1, soluble vascular cell adhesion molecule-1; t-tau, total tau; YKL-40, chitinase-3-like protein 1.

****Supplementary Figure 1. Associations between baseline core AD CSF biomarkers and change in Centiloid values at follow-up by sex

Scatter plots showing the associations between baseline core AD CSF biomarkers and change in Centiloid values at follow-up, stratified by sex. Each point represents an individual’s CSF biomarker value. Nominal *P*-values refer to the interaction term between sex and each CSF biomarker, adjusted for age, *APOE-*ε4 status and time difference between baseline and follow-up visits. Significant *P*-values are highlighted in bold. *P*-values were not significant after correction for multiple comparisons using FDR.

Supplementary Figure 2. Associations between baseline non-AD-specific CSF biomarkers and change in Centiloid values at follow-up by sex

Scatter plots showing the associations between baseline non-AD specific CSF biomarkers and change in Centiloid values at follow-up, stratified by sex. Each point represents an individual's CSF biomarker value. Nominal *P*-values refer to the interaction term between sex and each CSF biomarker, adjusted for age, *APOE*-ε4 status and time difference between baseline and follow-up visits. Significant *P*-values are highlighted in bold. *P*-values were not significant after correction for multiple comparisons using FDR.
